# Human CYP2B6 produces oxylipins from polyunsaturated fatty acids and reduces diet-induced obesity

**DOI:** 10.1371/journal.pone.0277053

**Published:** 2022-12-15

**Authors:** Melissa M. Heintz, Jazmine A. Eccles, Emily M. Olack, Kristal M. Maner-Smith, Eric A. Ortlund, William S. Baldwin

**Affiliations:** 1 Biological Sciences, Clemson University, Clemson, South Carolina, United States of America; 2 Emory Integrated Metabolomics and Lipodomics Core, Emory University, Atlanta, Georgia, United States of America; 3 Department of Biochemistry, Emory University School of Medicine, Emory University, Atlanta, Georgia, United States of America; Tokyo University of Agriculture, JAPAN

## Abstract

Multiple factors in addition to over consumption lead to obesity and non-alcoholic fatty liver disease (NAFLD) in the United States and worldwide. CYP2B6 is the only human detoxification CYP whose loss is associated with obesity, and Cyp2b-null mice show greater diet-induced obesity with increased steatosis than wildtype mice. However, a putative mechanism has not been determined. LC-MS/MS revealed that CYP2B6 metabolizes PUFAs, with a preference for metabolism of ALA to 9-HOTrE and to a lesser extent 13-HOTrE with a preference for metabolism of PUFAs at the 9- and 13-positions. To further study the role of CYP2B6 *in vivo*, humanized-CYP2B6-transgenic (hCYP2B6-Tg) and Cyp2b-null mice were fed a 60% high-fat diet for 16 weeks. Compared to Cyp2b-null mice, hCYP2B6-Tg mice showed reduced weight gain and metabolic disease as measured by glucose tolerance tests, however hCYP2B6-Tg male mice showed increased liver triglycerides. Serum and liver oxylipin metabolite concentrations increased in male hCYP2B6-Tg mice, while only serum oxylipins increased in female hCYP2B6-Tg mice with the greatest increases in LA oxylipins metabolized at the 9 and 13-positions. Several of these oxylipins, specifically 9-HODE, 9-HOTrE, and 13-oxoODE, are PPAR agonists. RNA-seq data also demonstrated sexually dimorphic changes in gene expression related to nuclear receptor signaling, especially CAR > PPAR with qPCR suggesting PPARγ signaling is more likely than PPARα signaling in male mice. Overall, our data indicates that CYP2B6 is an anti-obesity enzyme, but probably to a lesser extent than murine Cyp2b’s. Therefore, the inhibition of CYP2B6 by xenobiotics or dietary fats can exacerbate obesity and metabolic disease potentially through disrupted PUFA metabolism and the production of key lipid metabolites.

## Introduction

Obesity, in addition to insulin resistance and dyslipidemia, are the most important risk factors for development of nonalcoholic fatty liver disease (NAFLD) [1]. The prevalence of obesity is increasing in the United States, and the most recent 2017–2018 National Health and Nutrition Examination Survey recorded 42.4% of adults are obese [2]. Interstingly, reduced human hepatic CYP2B6 activity is associated with obesity [3]. Murine models also support a role for Cyp2b members as anti-obesity CYPs. The constitutive androstane receptor (CAR) has been identified as an anti-obesity transcription factor, and its activation in leptin-deficient mice induced cytochrome P450 2b10 (Cyp2b10) and subsequently improved hepatic glucose and fatty acid metabolism [4]. Additionally, a loss of hepatic CYP activity in hepatic P450 oxidoreductase (POR)-null mice led to steatosis and the induction of Cyp2b, including Cyp2b10, primarily through CAR activation [5], indicating a role for CAR in recognizing and Cyp2b in metabolizing hepatic lipids. Forkhead box protein A2 (Foxa2) regulates lipid metabolism and ketogenesis genes in mice including *Cyp2b9* [6, 7]. Three recent studies found *Cyp2b9* exhibited the highest increase in gene expression following a high-fat diet (HFD) in mice [8–10]. Our previously generated Cyp2b9/10/13-null (Cyp2b-null) mice are age- and diet-induced obese (DIO) with increased NAFLD in males [10, 11]. Similarly, age-dependent lipid accumulation was observed in RNAi-mediated Cyp2b-knockdown male mice [12]. These findings implicate Cyp2b in hepatic fatty acid metabolism and obesity.

CYP2B6 is the only hepatic CYP2B isoform in humans; *Cyp2b9*, *Cyp2b10*, and *Cyp2b13* are the dominant hepatic Cyp2b genes in mice. In the liver, CAR followed by pregnane X receptor (PXR) are the primary regulators of human and murine *Cyp2b* genes [13, 14]. CYP2B members are also regulated by the glucocorticoid receptor (GR) and FOXA2, hepatocyte nuclear factor 4α (HNF4α), and CCAAT/enhancer-binding protein α (C/EBPα) in humans and rodents [6, 15–18]. Growth hormone-mediated regulation of these transcription factors results in the female predominant expression of murine Cyp2b [17, 19]. Although there is large interindividual variation in human hepatic CYP2B6 expression, it is also primarily female expressed, but to a much lesser degree than rodents [20]. It is hypothesized that changes in the expression of transcription factors such as FOXA2 by fluctuations of steroids and hormones may contribute to the interindividual variations of CYP2B6 expression in human populations [15].

Human CYP2B6 has broad substrate specificity, playing a role in the metabolism of numerous xeno- and endobiotic compounds [21]. The substrate selectivity of CYP2B6 includes over 60 clinical drugs such as artemisinin, propofol, ketamine, ifosfamide, nevirapine, efavirenz, mephobarbital, bupropion, and tamoxifen [22], as well as many important environmental toxicants including chlorpyrifos [23], carbaryl [24], parathion [25], triclosan [26], perfluorocarboxylic acids [27], and the insect repellant *N*,*N*-diethyl-*m*-toluamide (DEET) [24]. Endogenous compounds such as steroids, bile acids, and fatty acids are also metabolized by CYP2B6 [22, 28].

Polyunsaturated fatty acids (PUFAs) may regulate Cyp2b transcription and act as Cyp2b substrates. For example, the omega-6 fatty acid, linoleic acid (LA), activates CAR and induces Cyp2b10 [5]. The omega-3 fatty acid, docosahexaenoic acid (DHA) inhibits CAR translocation and subsequently inhibits Cyp2b transcription [29]. Arachidonic acid is metabolized by Cyp2b19 (mouse) and CYP2B12 (rat) in keratinocytes, as well as CYP2B1/2 in rat hepatic microsomes to anti-inflammatory epoxyeicosatrienoic acids (EETs) [30–32]. Human CYP2B6 also appears to play a role in the epoxidation of anandamide, an arachidonic acid-derived endogenous cannabinoid to bioactive hydroxyeicosatetraenoic acid (HETE) and EET metabolites [33], such as 5,6-EET-ethanolamide, a potent agonist of the peripheral cannabinoid receptor, CB2 [34]. However, the role of human CYP2B6 in the metabolism of most PUFAs is not well-characterized or completely untested.

Murine Cyp2b enzymes are anti-obesogenic in males. Cyp2b-null mice are diet-induced obese with an increase in NAFLD, white adipose tissue, serum cholesterol, leptin, and β-hydroxybutyrate. Furthermore, Cyp2b-null males fed a normal diet show increased liver triglycerides and a gene expression profile similar to WT mice fed a HFD, indicating progression to NAFLD even without a high-fat diet [10]. In contrast, female weight gain was not significantly different, nor was NAFLD; conversely, the lack of Cyp2b in female mice was moderately protective from methionine and choline-deficient diet-induced non-alcoholic steatosis (NASH) [35]. However, the role of CYP2B6 as an anti-obesity gene has not been assessed. The purpose of this research is to determine the role of CYP2B6 in PUFA metabolism and test whether CYP2B6 is an anti-obesity enzyme by comparing diet-induced obesity (DIO) between Cyp2b-null mice and our newly produced humanized CYP2B6 mice to determine if human CYP2B6 can reverse obesity and NAFLD in Cyp2b-null mice. Physiological (body, tissue weights, glucose tolerance), biochemical (cholesterol, serum and liver lipids, PUFA metabolites), and transcriptomic (RNAseq) changes were measured. PUFA metabolites of CYP2B6 were identified in vitro from CYP2B6 containing baculosomes by LC-MS/MS and in vivo from serum and liver following high-fat diet treatment. Results indicate human CYP2B6 primarily metabolizes PUFAs in the 9- and 13- positions and partially reverses diet-induced obesity observed in Cyp2b-null mice potentially through peroxisome proliferator activated receptor (PPAR) activation, but with unexpected sexually dimorphic effects.

## Materials and methods

### CYP2B6 inhibition

The Vivid CYP2B6 Blue Screening kit with CYP2B6-containing baculosomes was obtained from ThermoFisher (Waltham, MA, USA) and used to screen for PUFA inhibition of CYP2B6 at PUFA concentrations from 0.001–100 μM. Nonylphenol, a known CYP2B6 inhibitor, was used as a positive control [36, 37]. Decreased fluorescence due to chemical inhibition was quantified on a Gen5 microplate reader (Synergy H1 Hybrid Reader, BioTek, Winooski, Vermont, USA) at 415/460 nm excitation/emission at 30-second intervals for 30 minutes in kinetic assay mode in accordance with manufacturer’s protocol. IC50 values were determined as described previously using GraphPad Prism 7.0 (Graphpad Software, San Diego, CA, USA) [38, 39]. Briefly, chemical concentrations were log10 transformed, sigmoidal concentration-response curves were fit using non-linear regression, log(inhibitor) vs normalized response—variable slope model with least squares ordinary fit. Confidence intervals were produced assuming asymmetrical distribution as recommended by GraphPad.

### CYP2B6 fatty acid substrates

CYP2B6 containing and control baculosomes^TM^ (Thermo Fisher) were incubated with 25 μM arachidonic acid (AA), linoleic acid (LA), α-linolenic acid (ALA), or docosahexaenoic acid (DHA) (n = 3) for two hours in VIVID^TM^ P450 reaction buffer and the NADPH-regeneration system (Thermo Fisher). Following incubation, samples were stored at -80°C and shipped on dry ice to the Emory Integrated Metabolomics and Lipidomics Core (EIMLC). Oxidized lipids were selectively extracted from the samples by solid phase extraction because of their low concentrations in comparison to other high abundance lipid species. This was done by depositing homogenized samples into a C18 solid phase extraction cassette, rinsing with hexane to remove nonpolar lipid species, and eluting with methyl formate. The recovered lipids were analyzed via LC-MS/MS in a multiple reaction monitoring (MRM) based method that selectively targets oxylipins using an AB SCIEX QTrap5500 enhanced high performance hybrid triple quadrupole/linear ion trap LC/MS/MS with a mass range of m/z 5 to 1250 in triple quadrupole mode and 5–1000 in LIT mode. The LC/MS/MS is paired with an ExionLC AC HPLC/UHPLC system with an ExionLC column oven and autosampler along with a computer workstation running LipidView software (AB SCIEX) [40, 41]. The concentration of the intracellular oxylipins were calibrated against external standards.

### High-fat diet treatment of Cyp2b-null mice and hCYP2B6-Tg

Animal care and associated procedures were approved by Clemson University’s Institutional Animal Care and Use Committee. *CYP2A13/CYP2B6/CYP2F1*-transgenic mice from Dr. Qing Yu and Dr. Xinxin Ding’s laboratory’s containing a bacterial artificial chromosome (BAC) of 210 kb from human chr19 containing *CYP2A13*, *CYP2B6*, *and CYP2F1* genes [42] were bred to Cyp2b9/10/13-null (Cyp2b-null) mice from our laboratory that lack the primarily hepatic murine Cyp2b members, *Cyp2b9*, *Cyp2b10*, and *Cyp2b13* [43] to produce humanized CYP2B6-transgenic (hCYP2B6-Tg) mice lacking the hepatic murine Cyp2b members [37]. These mice also express CYP2A13, which is primarily expressed in the nasal mucosa and lung and CYP2F1, which is primarily expressed in the lung [42]. Genotyping was performed by extracting genomic DNA isolated from tails or ear punches using the QuantaBio (Beverly, MA USA) AccuStart II Mouse Genotyping Kit according to the manufacturer’s instructions. Mice were typically genotyped within three weeks so that nerve endings were not fully formed in the tail and topical Emla cream was also used to as an anesthetic to reduce pain. Genotype was then confirmed using a three step PCR genotyping process. First, mice were genotyped to confirm that the Cyp2b9/10/13 cluster on murine chromosome 7 was deleted using the F2/R2 primer set: (F2: 5’-gccagggtcagcatattcaccaa-3’/ R2: 5’-gcacagacatcatgaggttctggtg-3’; 59°C), which produces an approximately 1100 bp fragment in the absence of these three Cyp2b members [43]. The absence of these three members was confirmed with a Cyp2b13 specific primer set (F1: 5’-cagactcttgttagaccggaccat-3’ / R1: 5’-ccccaaggaataaaattctacatg-3’; 59°C) that ensured the mice were not heterozygous [43]. hCYP2B6-2A13-Tg primer set (F1: 5’-cctggacagatgcctttaactccg-3’ / R1: 5’-tggctttgcacctgcctgact-3’; 63°C) then confirmed the presence of the human BAC clone containing CYP2B6 and the CYP2B6/2A13/2F1 P450 cluster on human chromosome 19 [42].

Cyp2b-null and hCYP2B6-Tg female and male mice (10 weeks old; n = 8 per sex) were fed a high-fat diet (HFD; Envigo TD.06414, 5.1 Kcal/g: 60.3% fat (37% saturated, 47% monounsaturated, 16% polyunsaturated fat), 18.4% protein, 21.3% carbohydrates; Madison, WI USA) for 16 weeks. The lipid composition is defined and contains 3.1g/kg myristic acid (14:0), 76.6 g/kg palmitic acid (16:0), 41.5 g/kg stearic acid (18:0), 6.2 g/kg palmitoleic acid (16:1), 127.8 g/kg oleic acid (18:1), 71.7 g/kg linoleic acid (18:2), and 5.5 g/kg alpha-linolenic acid (18:3). Weight gain was monitored weekly and feed consumption was measured every other day. Glucose tolerance tests (GTT) were performed during week 13. At the end of the study, mice were anesthetized with isoflurane and blood collected by heart puncture prior to euthanasia confirmed by carbon dioxide apyxiation and bilateral pneumothorax. Liver, kidney, inguinal white adipose tissue (WAT), brown adipose tissue (BAT), and testes were excised and weighed. All tissues were immediately snap frozen with liquid nitrogen and stored at -80°C.

### Fasting blood glucose and glucose tolerance tests

Mice were fasted for 4.5 hours and fasting blood glucose was determined using an Alphatrak 2 (AlphaTRAK, Chicago, IL USA) blood glucose meter following tail bleed on week 13. Then glucose tolerance was determined following an intraperitoneal injection of 1g/kg D-glucose (Sigma Ultra, St. Louis, MO USA) with blood glucose readings from tail bleeds every 20 min for the first hour and every 30 min for the second hour as described previously [10, 44]. Results are recorded over the time and as area under the curve (AUC). Data are presented as mean blood glucose levels ± SEM. Statistical significance was determined by unpaired Student’s t-tests using GraphPad Prism 7.0.

### Serum biomarker panel

Serum parameters were measured as described previously [12]. Blood samples were collected by heart puncture and incubated at room temperature for 30 min followed by centrifugation at 6000 rpm for 10 min. Serum from each sample was transferred into a fresh tube and aliquots shipped on dry ice to Baylor College of Medicine’s Comparative Pathology Laboratory (Houston, TX USA) for determination of serum biomarker concentrations including alanine aminotransferase (ALT), cholesterol, triglycerides (TAG), high density lipoprotein (HDL), low density lipoprotein (LDL), and very low density lipoprotein (VLDL). Serum parameters were determined using a Beckman Coulter AU480 chemistry analyzer (Atlanta, GA, USA) and the appropriate Beckman Coulter biochemical kits according to the manufacturer’s instructions.

### Liver triglycerides

Liver triglycerides were extracted and quantified as described previously [45] using colorimetric kits from Cayman Chemical (Ann Arbor, MI). In addition, visual confirmation was performed with Oil Red O. During necropsy, clean liver slices were snap frozen in liquid nitrogen and stained with Oil Red O at Baylor College of Medicine’s Comparative Pathology Laboratory using standard protocols [4].

### Lipidomic analysis of polyunsaturated fatty acid (PUFA) metabolites

Serum and liver samples were shipped on dry ice to EIMLC for lipidomic analysis of lipid metabolites from AA, LA, ALA, DHA, and eicosapentaenoic acid (EPA). Oxidized lipids were selectively extracted from samples by solid phase extraction following EIMLC protocols [41].

Random forest analyses by MetaboAnalyst 3.6 [46] were performed on lipidomic data to rank PUFA metabolite species as a prediction of how large of an effect each species has between genotype [47]. The mtry parameter was set to 7, and the number of trees to be built was set to 500 for each analysis to achieve the lowest out-of-bag (OOB) error. The larger the mean decreased accuracy (MDA) value, the more important the lipid metabolites are for the accuracy of the association between variable and response. Lipid metabolites with importance scores less than or equal to zero are likely to have no predictive ability. Statistical significance between genotypes was also determined by unpaired Student’s t-tests using GraphPad Prism 7.0. Data are presented as mean concentration ± SEM.

### PPAR transactivation assays

PUFA and oxylipin PPAR agonist activity was measured (n = 2–3) using commercially available (Indigo Biosciences, State College, PA) murine and human PPARα (NR1C1), PPARδ (NR1C2), and PPARγ (NR1C3) reporter assay systems according to the manufacturer’s directions. Oxylipins were purchased from Cayman Chemical Co (Ann Arbor, MI).

### RNA sequencing (RNAseq)

Liver samples were stored in RNAlater Stabilization Solution (Invitrogen, Carlsbad, CA USA) at -80°C. Total RNA was extracted from mouse livers of each treatment group using TRIzol (Ambion, Carlsbad, CA USA) and quantified on a Qubit 2.0 Fluorometer. RNA integrity number (RIN) was determined with an Agilent 2100 Bioanalyzer (place) to assess RNA quality, and samples with a RIN > 8.0 were determined to be of high quality and used for next generation sequencing. Libraries were prepared using NEB Next Ultra RNA Library Prep kit. Samples were sequenced to an average sequencing depth of 20,000,000 read pairs with a 2x150 paired-end module using a NovaSeq 6000. Quality metrics were checked using FastQC on all samples sequenced, and Trimmomatic was used to trim low quality bases. Trimmed reads were aligned to the *Mus musculus* reference genome (GCF_000001635.25_GRCm38.p6) using GSNAP, and 100% of the trimmed reads aligned. Subread feature counts software found reads that aligned with known genes. Raw read counts and EdgeR were used to determine differential gene expression [48]. Series GSE148460 containing the RNAseq data has been uploaded to the Gene Expression Omnibus (GEO).

DAVID functional annotation tool was used to perform the analysis of enriched gene ontology (GO) terms from differentially expressed gene lists for female and male mice (adjusted p-value < 0.05) [49]. Chord plots were generated in R using GOplot to display the relationship between enriched GO terms and differentially expressed genes. Hierarchical cluster analysis was performed on variables including total body weight, WSI, serum lipids, oxylipin species from liver and serum, and differentially expressed genes (logFC > 1.0 or < 1.0) and visualized in heatmaps with MetaboAnalyst 3.6 [46] to compare measured variables between genotypes. MAGIC was used to estimate transcription factors involved in differential gene regulation [50], and Enrichr was used to compare our differential gene expression set to other transcriptomic gene sets [51].

### Quantitative real-time PCR (qPCR)

RNA (2 μg) was used to prepare cDNA with 10 mM dNTPs, 200 units MMLV reverse transcriptase, and 50 μg random hexamers (Promega, Madison WI, USA). Primers used were previously published [37, 45] and pimer sequences and annealing temperatures are provided in Table 1. Samples were diluted 1:10 and amplified in triplicate using a 96-well CFX Real-Time PCR (Bio-Rad, Hercules CA, USA) with 0.25X RT^2^ SybrGreen (Qiagen, Frederick MD, USA) compared to the geometric mean of the reference genes, 18S and GAPDH. Standard curves were used to determine efficiency using a mix of samples containing all cDNA samples diluted at 1:1, 1:4, 1:16 1:64, 1:256, and 1:1024 and gene expression quantified using the modified Muller’s method [52, 53].

**Table 1:** Primer sequences for determining hepatic gene expression of insulin, Srebf, and Ppar regulated genes involved in lipid uptake, storage, and metabolism.

Gene	Forward sequence	Reverse sequence	Annealing temperature (°C)
Gapdh	CCTTCATTGACCTCAACTA	CTGGAAGATGGTGATGG	50
18S	ATGGCCGTTCTTAGTTGGTG	ATGCCAGAGTCTCGTTCGTT	64
Fasn	ATTGCATCAAGCAAGTGCAG	GAGCCGTCAAACAGGAAGAG	54.2
Pparg	TGGGTGAAACTCTGGGAGATTC	AATTTCTTGTGAAGTGCTCATAGGC	60.1
Ppard	ATCCTCACCGGCAAGTCCA	CCTGCCACAGTGTCTCGATG	60
Cyp4a14	GAGCCGTCAAACAGGAAGAG	GAGTCCATAGGCCTGAGTTATTT	59
Pepck1	GTCAACACCGACCTCCCTTA	CCCTAGCCTGTTCTCTGTGC	60.6
Srebf1	ACGAAGTGCACACAAAAGCA	GCCAAAAGACAAGGGGCTAC	58
Cd36	GCTTGCAACTGTCAGCACAT	GAGCTATGCAGCATGGAACA	60
Cpt1a	TTGATCAAGAAGTGCCGGACGAGT	GTCCATCATGGCCAGCACAAAGTT	60

## Results

### Inhibition of CYP2B6 by endogenous compounds

**Fig 1:**
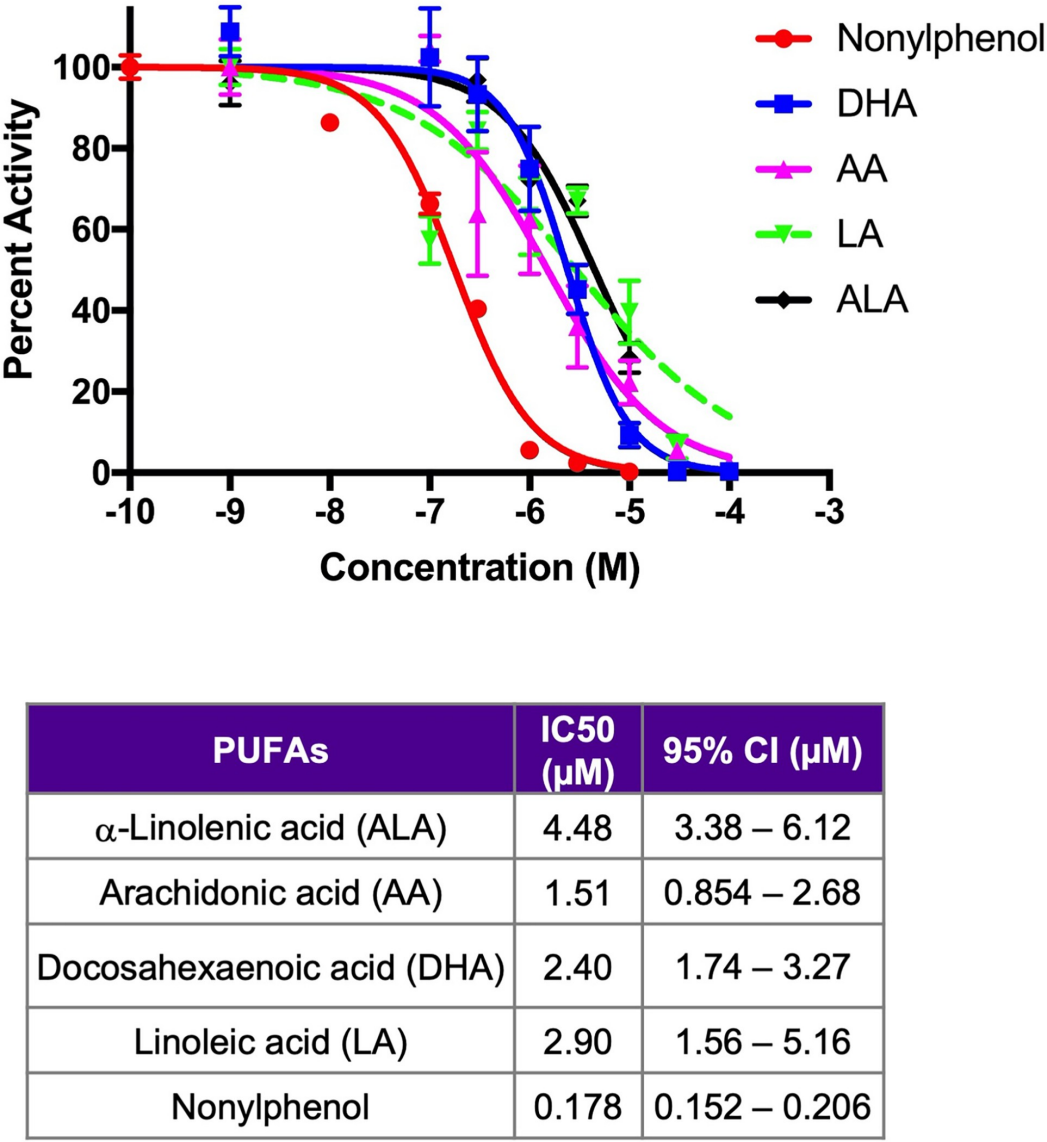
Fatty acid inhibitors of CYP2B6. Concentration-response curves of CYP2B6 containing baculosomes treated with polyunsaturated fatty acids (PUFAs) or nonylphenol (positive control). Data are generated as described in the Materials and Methods using GraphPad Prism 7.0 and presented as mean ± SEM in the graph with IC50s and 95% Confidence Intervals included.

The concentration-dependent inhibition of the PUFAs AA, ALA, DHA, and LA were determined using CYP2B6 containing baculosomes (**Fig 1**) with the plasticizer, nonylphenol, as a positive control [37]. In concurrence with recently performed screening results of multiple endobiotic and xenobiotic inhibitors [36], AA and DHA had the lowest IC50s (1.51 μM and 2.40 μM, respectively) compared to the other PUFAs, LA (2.90 μM) and ALA (4.48 μM) (**Fig 1**). It should be noted that all of the PUFAs had overlapping 95% CI except AA and ALA. However, even the strongest endogenous inhibitors have almost 10X times less affinity / inhibitory capacity to CYP2B6 than the known xenobiotic inhibitor, nonylphenol [37]. Because of dietary sources the PUFAs probably reach much greater hepatic concentrations than xenobiotics and therefore it is expected that these PUFAs are effective inhibitors and potential substrates for CYP2B6; at least following a HFD or a diet high in these PUFAs. Percent inhibition of CYP2B6 along with IC50s and Hillslopes by multiple PUFAs, pesticides, and other xenobiotics in comparison to CYP3A4 was recently published and available for comparison [36].

### Preferential metabolism of ALA to 9-HOTrE by CYP2B6-containing baculosomes

**Fig 2:**
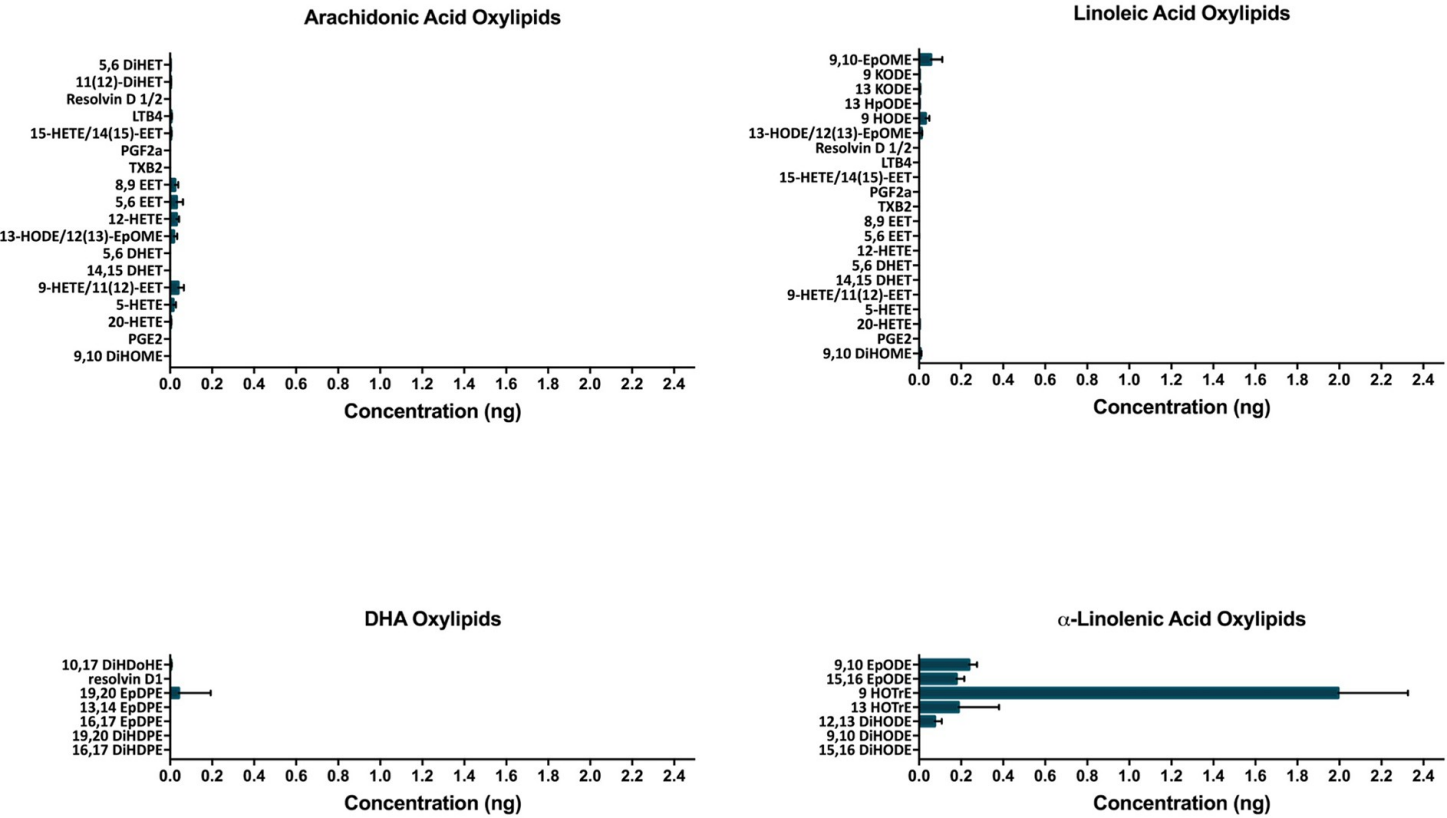
PUFA metabolites of CYP2B6. LC-MS/MS was used to measure production of oxylipid metabolites formed by CYP2B6 containing baculosomes compared to control baculosomes incubated with 25 μM arachidonic acid, linoleic acid, docosahexaenoic acid, and α-linolenic acid (n = 3).

Oxylipins of AA, LA, DHA, and ALA produced by CYP2B6 were measured by LC-MS/MS to further investigate the role of CYP2B6 in PUFA metabolism. Surprisingly few AA, LA and DHA metabolites were formed considering their IC50s (**Fig 2**). Instead, the n-3 PUFA, ALA, was the most prominently metabolized PUFA with metabolite concentrations almost 20X greater than other PUFA metabolites. 9-hydroxy-10E,12Z,15Z-octadecatrienoic acid (9-HOTrE) and 13-HOTrE were the primary oxylipins produced. Such high concentrations suggest that CYP2B6 has a specific PUFA substrate and products, potentially as key signaling molecules. Interestingly, in addition to ALA, other n-3 and n-6 based oxylipins were also primarily metabolized at the 9 or 13 position, indicating that CYP2B6 preferentially metabolizes PUFAs, such as ALA and LA, in these positions.

### Humanized-CYP2B6-Tg mice have increased glucose sensitivity and decreased body mass in comparison to Cyp2b-null mice following 16 weeks of HFD treatment

**Fig 3:**
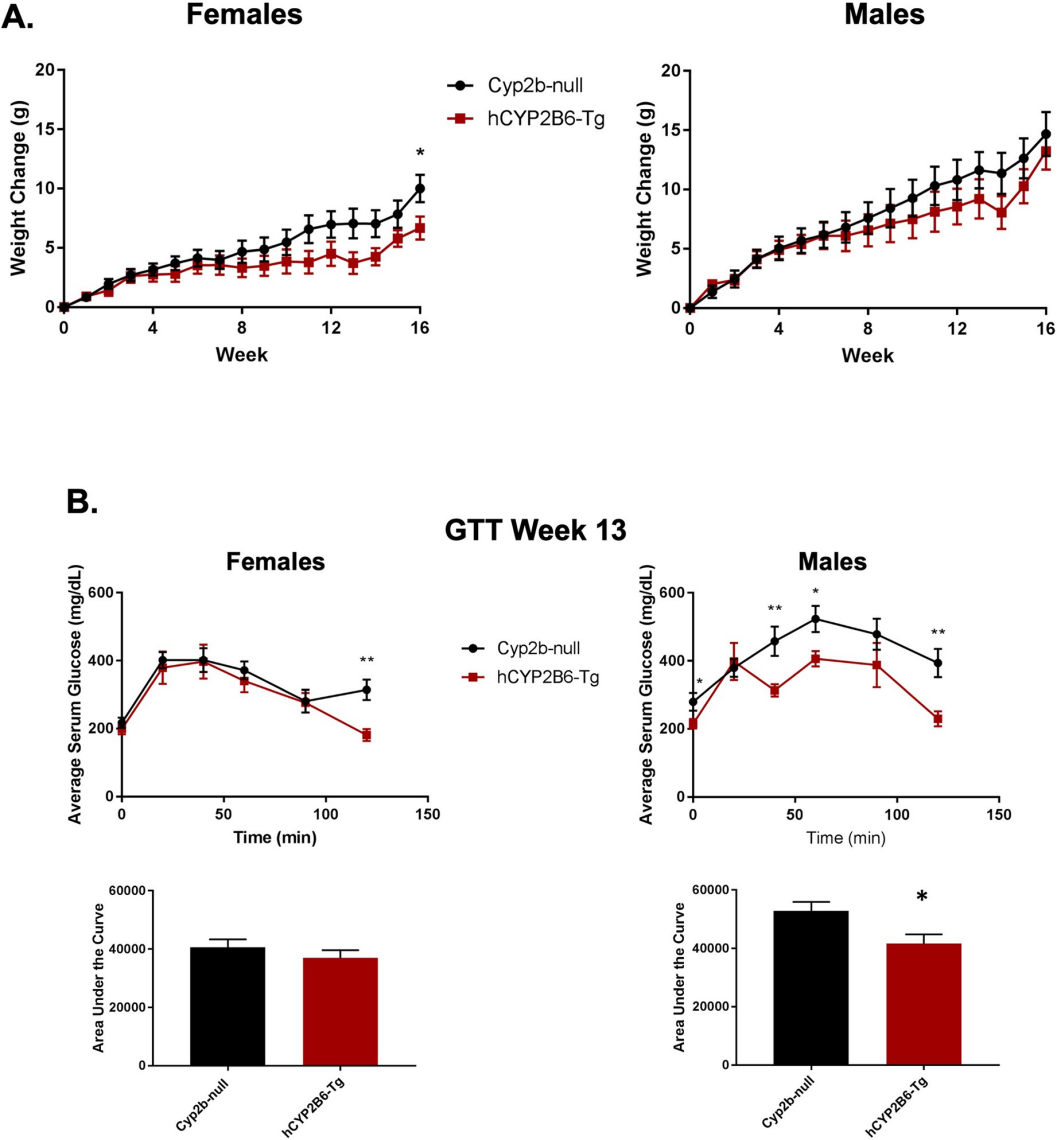
Genotypic differences in body weight gain and glucose sensitivity during 16 weeks of high-fat diet treatment. (A) Change in body weight gain over 16 weeks of HFD treatment. (B) Glucose tolerance tests (GTT) performed during week 13 on Cyp2b-null and hCYP2B6-Tg female and male mice. Results are represented as area under the curve. Data are presented as mean ± SEM. Statistical significance was determined by unpaired Student’s t-tests (n = 7–8). * indicates a p-value < 0.05 and ** indicates a p-value < 0.01.

Female hCYP2B6-Tg mice gained significantly less weight than their Cyp2b-null counterparts after 16 weeks of a HFD. Changes in body mass were similar until about week 11 and continued to separate by genotype for the duration of the study (**Fig 3A**). In contrast, male hCYP2B6-Tg mice showed no significant differences in body mass compared to Cyp2b-null mice over the 16 weeks of HFD treatment; although male hCYP2B6-Tg mice tended to gain a little less weight than their Cyp2b-null counterparts during the second half of the study (**Fig 3A**). Both genotypes in female and male mice consumed similar amounts of calories throughout the duration of the 16-week HFD study (**S1 File**); therefore, caloric consumption does not explain the differences in body mass. The reduced weight gain in hCYP2B6-Tg female mice may be partly attributed to lower tissue weights as inguinal WAT was decreased 27% although not significantly; only kidney weights were significantly decreased (**S2 File**). No differences were observed in tissue weights between Cyp2b-null and hCYP2B6-Tg male mice (**S2 File**).

GTTs were performed to determine if HFD-fed hCYP2B6-Tg mice respond better than HFD-fed Cyp2b-null mice to a glucose challenge, a biomarker of metabolic disease. Female hCYP2B6-Tg mice performed slightly better than their Cyp2b-null counterparts, but only at the last time point, suggesting that this may be an outlier. However, male hCYP2B6-Tg mice exhibited a significantly faster response to a glucose challenge than Cyp2b-null males even though there was no change in weight between the genotypes (**Fig 3B**), suggesting some other physiological change is protecting hCYP2B6-Tg mice from metabolic disease.

### Hepatic and serum lipids in HFD-fed hCYP2B6-Tg mice

**Fig 4:**
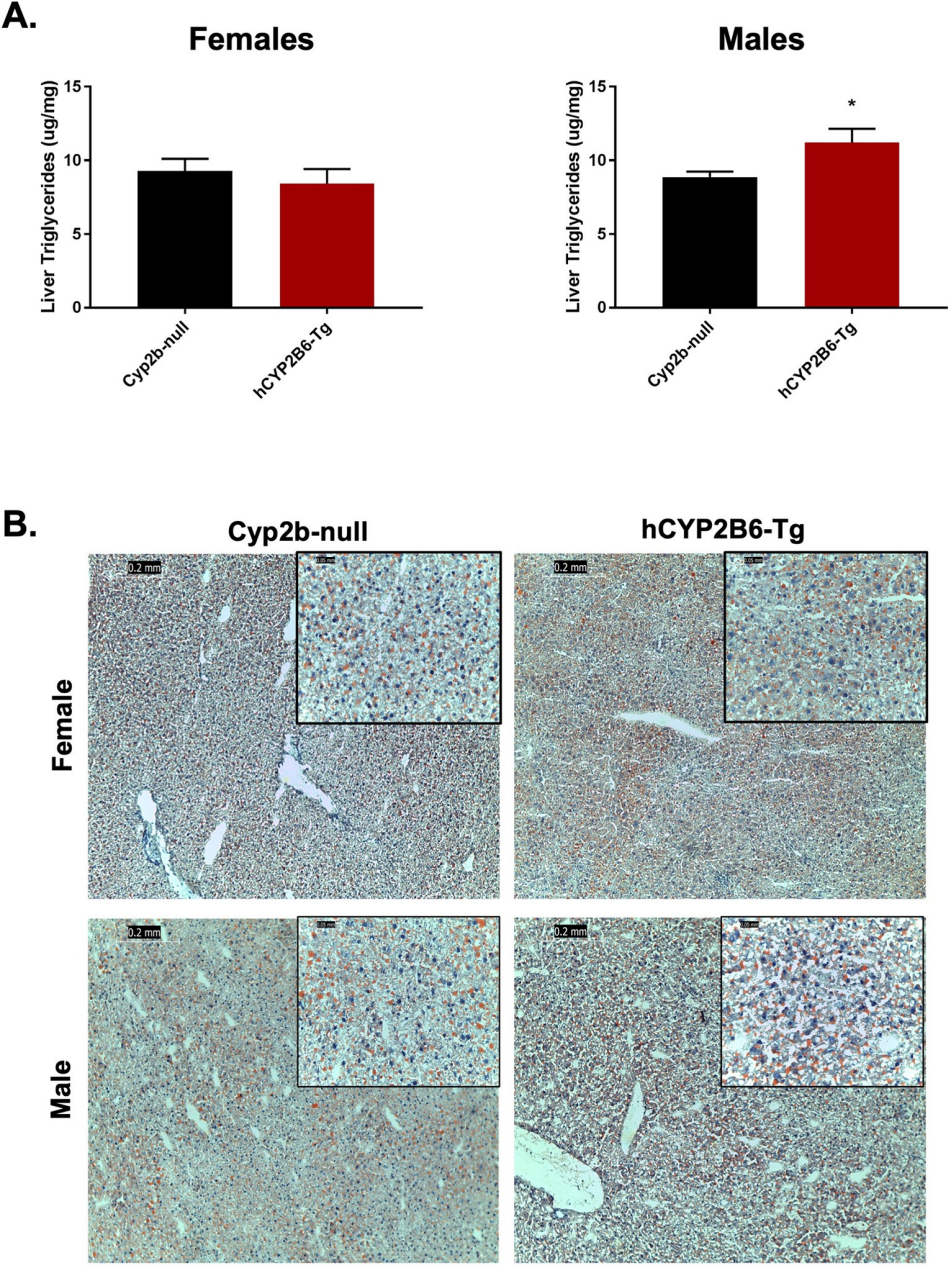
Comparison of steatosis markers in hCYP2B6-Tg and Cyp2b-null mice. (A) Total liver triglycerides were measured in Cyp2b-null and hCYP2B6-Tg female and male mice. Data are presented as mean + SEM. Statistical significance was determined by unpaired Student’s t-tests (n = 5). * indicates a p-value < 0.05. (B) Fatty liver histopathological changes were evaluated by Oil red O staining in female and male mice. Images were taken at 100x (0.2 mm) and 400x (0.05 mm) magnification.

Differences in hepatic lipid accumulation between Cyp2b-null and hCYP2B6-Tg mice were examined to determine if the presence of hCYP2B6 provided protection from NAFLD as do murine Cyp2b members in male mice [5, 10]. Surprisingly, male but not female hCYP2B6-Tg mice showed increased hepatic triglycerides compared to Cyp2b-null mice (**Fig 4A**); the opposite of what was predicted and observed in wildtype compared to Cyp2b-null male mice [10]. Histological analysis of Oil Red O staining was performed to confirm the chemical analysis of lipid accumulation in the liver. Although steatosis increased as a result of HFD treatment, differences observed between genotypes were relatively small when confirmed by Oil Red O staining (**Fig 4B**). There were also no significant differences in serum lipids between Cyp2b-null and hCYP2B6-Tg mice (**S3 File**). Overall, the increase of inert liver triglycerides in hCYP2B6-Tg mice may paradoxically be protective based on increased glucose sensitivity although other measured physiological and biochemical parameters are equivocal [54, 55].

### Hepatic and serum oxylipin metabolites produced in HFD-fed hCYP2B6-Tg mice

**Fig 5:**
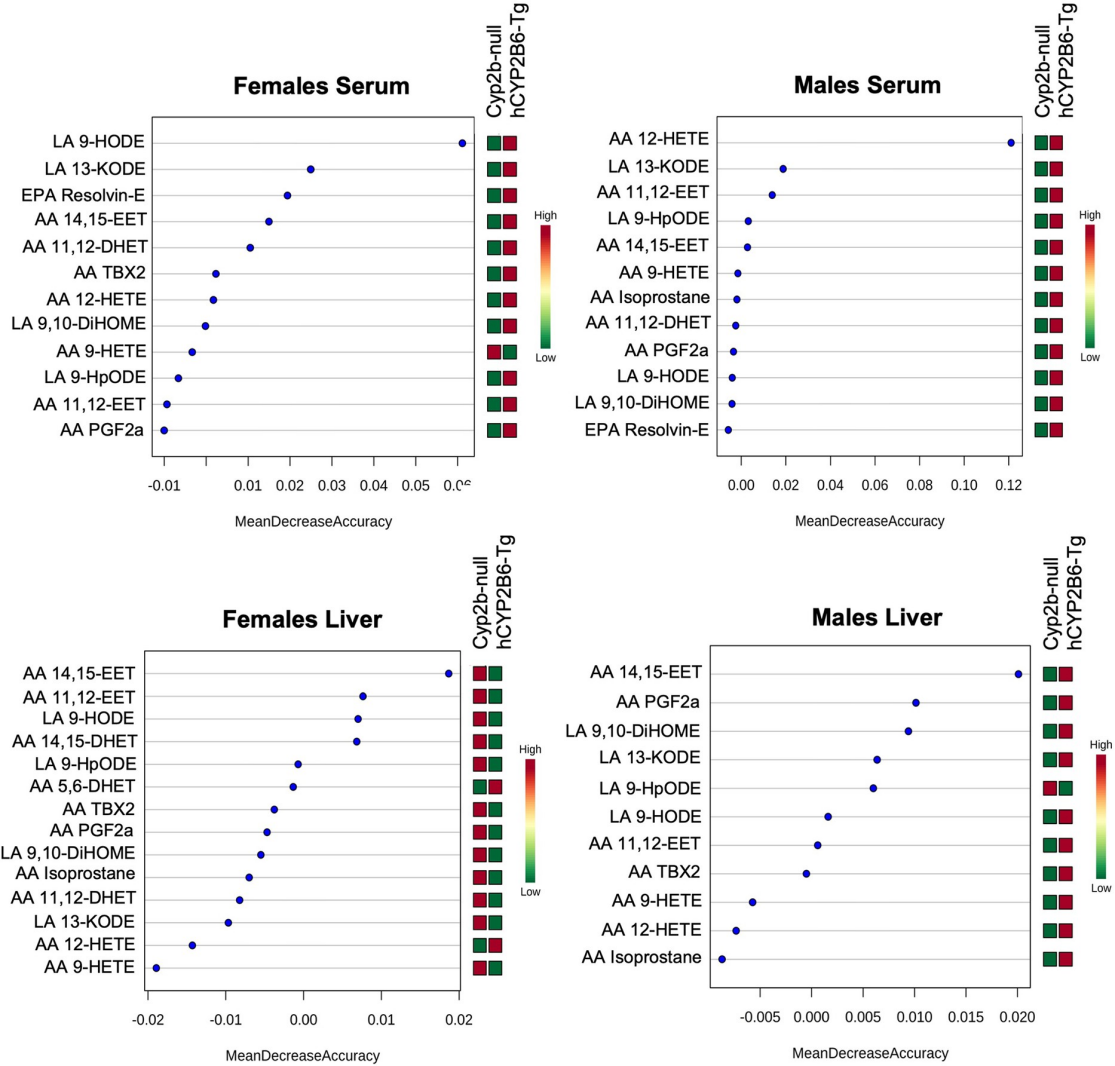
Random forest analysis of lipid metabolites by tissue type in female and male mice. Important lipid metabolites identified by random forest analysis between Cyp2b-null and hCYP2B6-Tg mice in serum or liver tissue.

Concentrations of serum and hepatic lipid metabolites from LA, AA, ALA, DHA, and EPA were compared between HFD-fed Cyp2b-null and hCYP2B6-Tg mice to identify lipid metabolites metabolized by human CYP2B6 *in vivo*. The oxylipin species most predictive of differences between Cyp2b-null and hCYP2B6-Tg mice in serum or liver tissue are primarily AA and LA-species as determined by random forest analysis and are almost always produced at higher concentrations in the hCYP2B6-Tg mice, presumably due to the presence of CYP2B6 (**Fig 5**).

The anti-inflammatory signaling molecule, AA 14,15-EET was ranked the most predictive lipid metabolite in hepatic tissue in female and male hCYP2B6-Tg mice (**Fig 5**). Interestingly, AA 14,15-EET was the only statistically different oxylipin by unpaired Student’s t-tests between Cyp2b-null and hCYP2B6-Tg female mice in liver, and there were no significant changes in oxylipin concentrations between HFD-fed Cyp2b-null and hCYP2B6-Tg mice in the liver. While individual hepatic oxylipin differences were rarely significant, the total average concentration of oxylipins in the liver significantly increased in male hCYP2B6-Tg mice (**S4 File**).

Hepatic pro-inflammatory response metabolites, LA 9-hydroxyoctadecadienoic acid (LA 9-HODE) (females) and AA 12-hydroxyeicosatetraenoic acid (AA 12-HETE) (males), followed by LA 13-HODE derivative, 13-keto-9Z,11E-octadecadienoic acid (LA 13-KODE), were identified as the most predictive lipid metabolites in serum of female and male mice (**Fig 5**). With the exception of AA 12-HETE, these metabolites and others were also different by Student’s t-tests (**S5 and S6 Files**). LA 9,10-dihydroxyoctadec-12-enoic acid (DiHOME) a hydrolase metabolite of 9,10-epoxy-12Z-octadecenoic acid (EpOME), LA 9-HODE and LA 13-KODE, as well as AA 14,15-epoxyeicosatrienoic acid (AA 14,15-EET) and ALA isoprostane all increased in the serum of female hCYP2B6-Tg mice (**S5 File; Fig 5)**. These PUFA metabolites also increased in the serum of male hCYP2B6-Tg mice but not significantly. The only lipid metabolite to significantly increase in the presence of CYP2B6 in the serum of male mice was AA thromboxane B2 (TXB2) (**S5 File**). Total average oxylipin concentrations in serum samples increased, but not significantly, in both female and male hCYP2B6-Tg mice compared to Cyp2b-null mice (**S4 File**).

### Gene expression differences between HFD-fed Cyp2b-null and hCYP2B6-Tg mice

**Fig 6:**
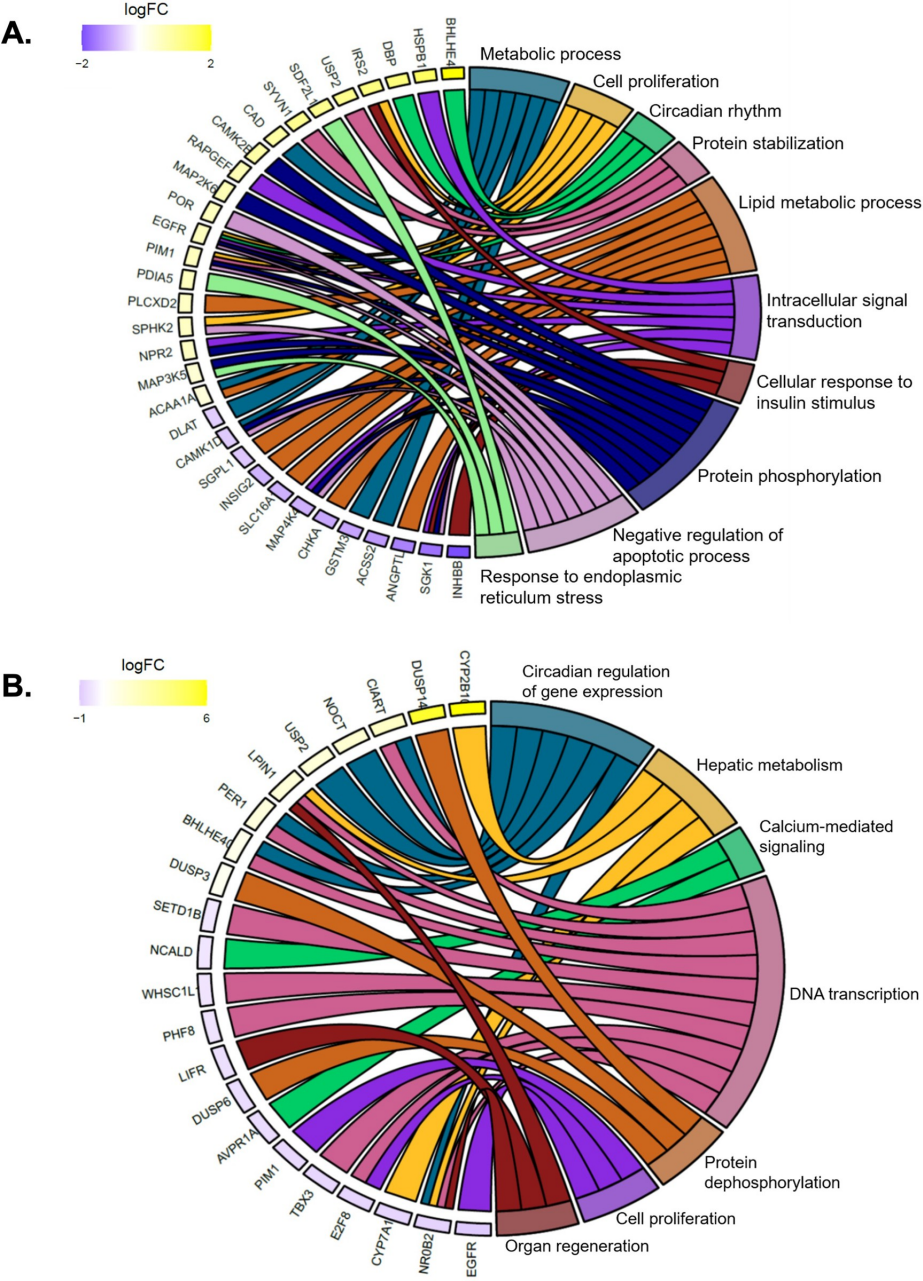
Chord plots displaying relationships between RNAseq gene expression data and gene ontology. Chord plots were used to display the relationship between enriched GO terms and differential gene expression data in female (A) and male (B) mice.

RNAseq was performed on liver samples to examine the effect of CYP2B6 on global hepatic gene expression compared to Cyp2b-null mice during HFD treatment (**S7 File**). Gene ontology (GO) enrichment chord plots were used to display the relationship between differentially expressed genes and enriched biological process GO terms (**S7 File**). Several circadian rhythm-associated genes were up-regulated in HFD-fed hCYP2B6-Tg mice compared to Cyp2b-null mice (**Fig 6**). Circadian regulation plays an important role in liver metabolism and metabolic disease [56]. Female and to a lesser extent, male hCYP2B6-Tg mice had several perturbed protein processing and phosphorylation associated genes. Previous RNAseq data generated in our lab determined Cyp2b-null mice have increased endoplasmic reticulum stress compared to wild-type mice [10]. Consistent with these results, female and to a lesser extent, male hCYP2B6-Tg mice had several perturbed protein processing and phosphorylation associated genes compared to Cyp2b-null mice. In addition, female hCYP2B6-Tg mice had several down-regulated genes involved in lipid synthesis, notably *Angptl8*, a critical modulator of serum triglyceride levels [57] (**Fig 6**).

Interestingly in males, *Cyp2b10* was the second highest induced gene (logFC = 6.19) in HFD-fed hCYP2B6-Tg compared to Cyp2b-null mice (**S7 File**). Cyp2b10 is the murine ortholog to human CYP2B6 and 74% identical. Based on alignment program settings and sequencing variation (i.e. number of SNPs), CYP2B6 reads were aligned to Cyp2b10 on the reference genome (**S8 File**). Furthermore, the epidermal growth factor receptor (*Egfr*) was down-regulated (logFC = -1.34) in male hCYP2B6-Tg mice compared to Cyp2b-null mice. The down-regulation of EGFR provides a mechanism for the activation of the constitutive androstane receptor (CAR) and subsequent induction of Cyp2b10/CYP2B6 [58]. Conversely, *Cyp2b10* was not differentially expressed in females, probably because *Egfr* was up-regulated (logFC = 0.77) in female hCYP2B6-Tg mice.

Our differentially expressed gene set was compared to others using Enrichr [51] with similarities most strong to CAR-null > PPARa-null = AhR-null > BHLHA15-null = HNF1-null > STAT3-null = ESRRA-null mice. Several different experiments with CAR-null mice appeared indicating that the gene expression patterns observed were most closely related to CAR-null mice with PPARa and AHR-null mice not far behind. Individual genes were run through MAGIC [50] to verify the results of Enrichr. The three transcription factors that regulate the most differentially expressed genes are FoxA2, PPARs, and sterol regulatory element binding transcription factor 1 (SREBF1). ESRRA and NRF2 were also represented. Estrogen related receptors (ESRRA) are often associated with PPAR related processes [59]. CAR was not well represented in MAGIC and even CYP2B6 did not show CAR regualation in MAGIC. PPARgene confirmed the regulation of several genes by the PPARs [60].

**Fig 7:**
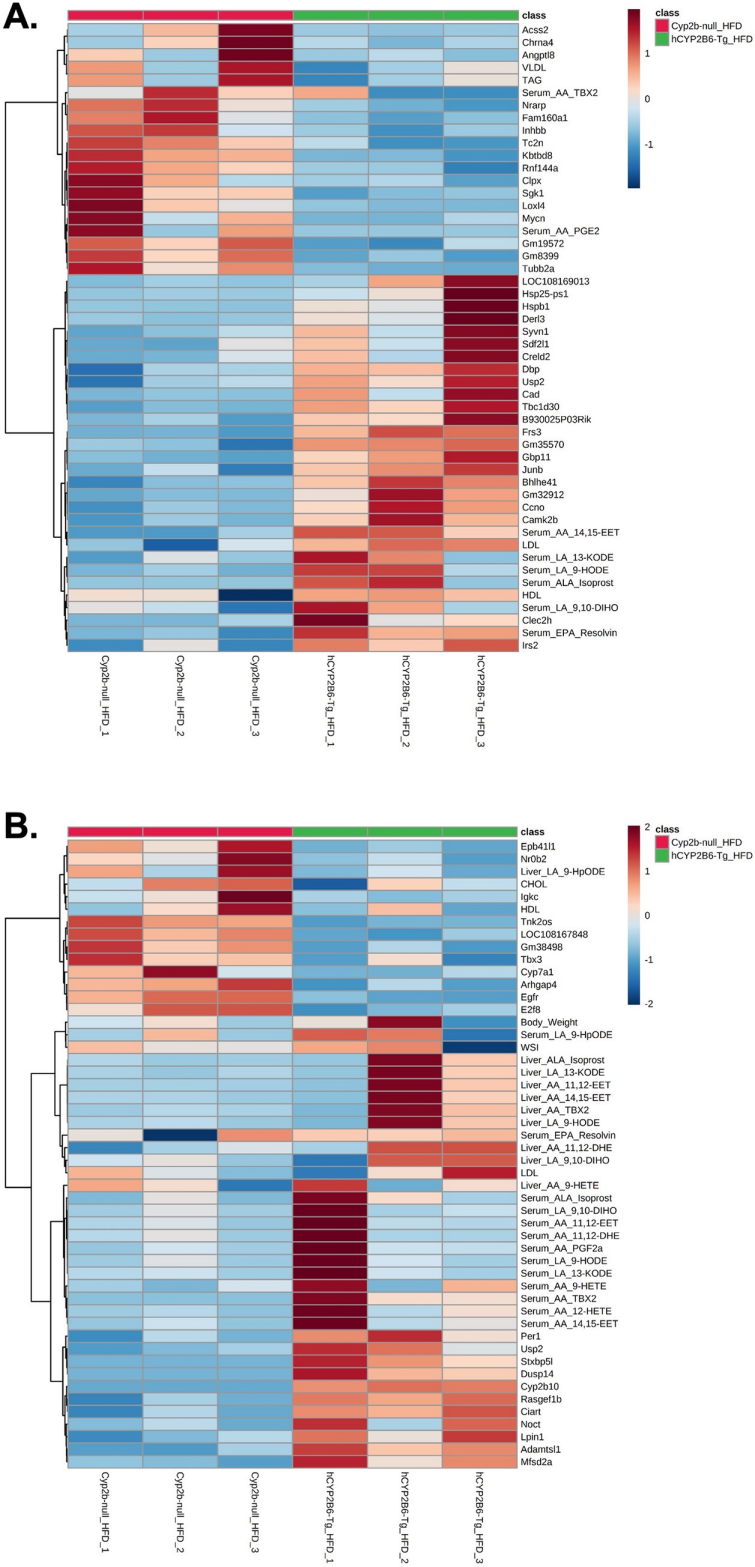
Hierarchical cluster analysis comparing measured variables between HFD-fed hCYP2B6-Tg and Cyp2b-null mice. Hierarchical cluster analysis determining the top 50 measured variables associated with HFD-fed Cyp2b-null or hCYP2B6-Tg female (A) or male (B) mice visualized in heatmaps. Variables include serum lipids, WAT somatic index (WSI), body weight, serum and liver oxylipin species, and differentially expressed genes (logFC >1.0 or <1.0).

To determine associations between physiological parameters, serum lipids, serum and liver oxylipin species, and differentially expressed genes between HFD-fed Cyp2b-null and hCYP2B6-Tg mice, hierarchical cluster analysis was performed using the top 50 measured variables from female and male mice (**Fig 7**). There was clear separation by genotype in females, with hierarchical clustering showing that in addition to differentially expressed genes, serum-associated variables make up the most distinguishing factors between genotypes in female mice (**Fig 7A**). HFD-fed hCYP2B6-Tg female mice were associated with an increase in several serum oxylipins, LDL, and HDL as well as more differentially expressed genes than Cyp2b-null females. Fewer serum parameters, TAG, VLDL, and two AA serum oxylipin species (AA-TBX2; AA-PGE2) (role in inflammation) were increased in HFD-fed Cyp2b-null female mice (**Fig 7A**).

Samples also clustered by genotype in male mice, but with more variability than females and a clear indication that some serum and liver oxylipin concentrations were inversely related (**Fig 7B**). Interestingly, HFD-fed Cyp2b-null males were associated with an increase in serum cholesterol and HDL and only one liver oxylipin metabolite. Conversely, hCYP2B6-Tg counterparts were correlated with an increase in WSI, LDL, and numerous serum and liver oxylipin species (**Fig 7B**).

**Fig 8:**
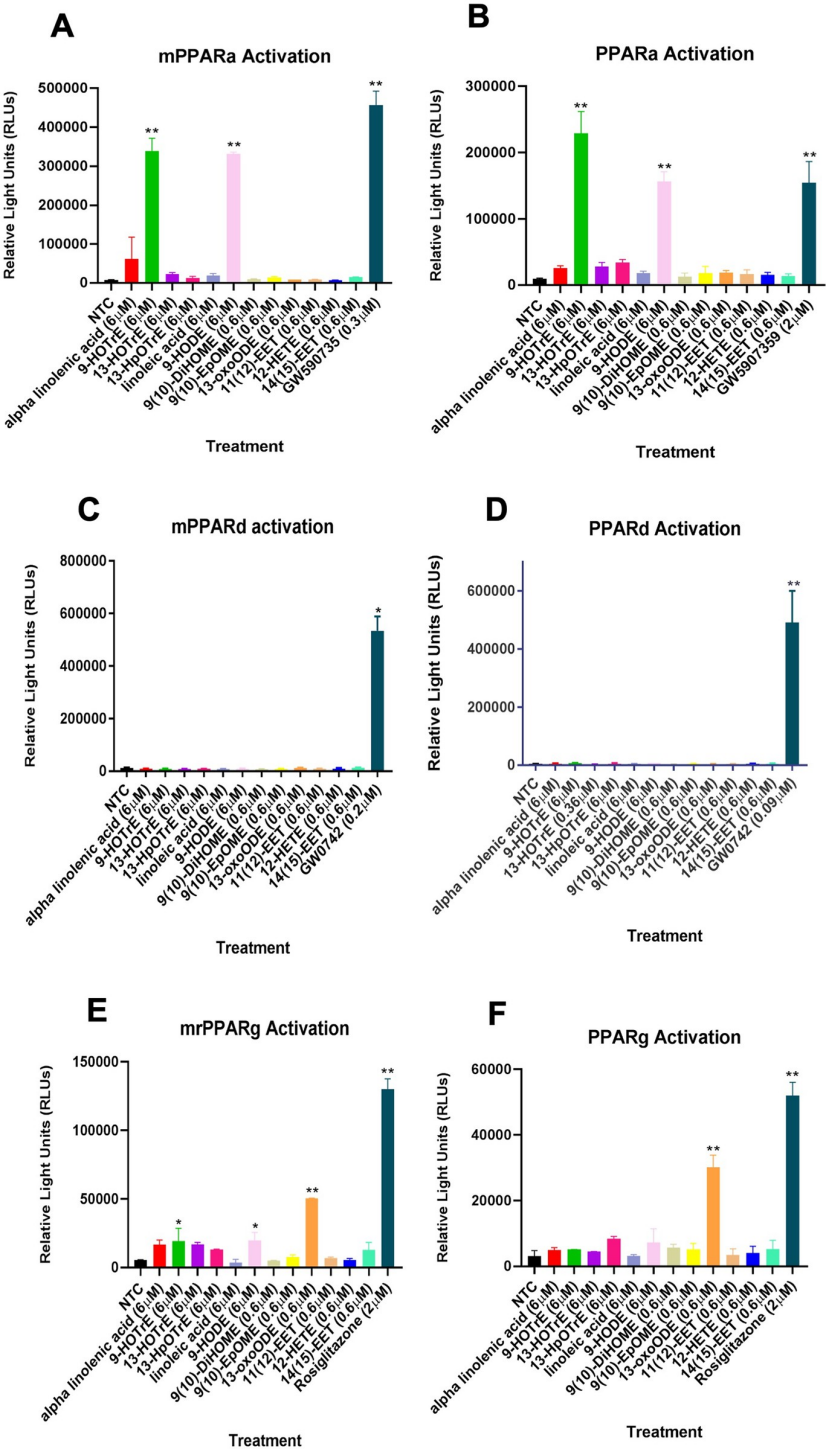
Transactivation assays reveal some CYP2B6-produced oxylipins activate PPARs. Mouse and human (A/B) PPARα, (C/D) PPARδ, and (E/F) PPARγ are activated by oxylipins primarily oxidized in the 9 and 13 positions. Data are presented as mean ± SEM. Statistical significance was determined by unpaired Student’s t-tests (n = 2–3). * indicates a p-value < 0.05 and ** indicates a p-value < 0.01.

Overall, this data indicated the importance of changes in expression and activity of nuclear receptors with CAR, PPARα, PPARγ, Rev-Erbβ, GR, ESSRA, and SHP all as potential contributors to changes in the regulation of key genes involved in circadian rhythms, cholesterol and lipid metabolism in female and male mice based on the literature (**Fig 7**)[61–63]. Because PPARs are involved in hepatic circadian rhythms, lipid metabolism, previous data indicates PPARγ activation by the oxylipin 13-KODE [64], and PPARs are common targets based on the Enrichr and MAGIC databases, we evaluated PPARα/δ/γ activation by oxylipins produced by CYP2B6. The primary oxylipins produced in vitro and in vivo in the 9-positions from linolenic and linoleic acid (9-HOTre and 9-HODE, respectively) by CYP2B6 are strong PPARα activators at 6 μM (**Fig 8A and 8B**). None of the oxylipins tested activated PPARδ (**Fig 8C and 8D**). 13-KODE, (also known as 13-oxo-ODE) moderately activated PPARγ at a relatively low concentration of 0.6 μM (**Fig 8E and 8F**), confirming previous results [64]. This work indicates that the oxylipins produced by CYP2B6 could activate PPAR pathways crucial in the distribution and utilization of fatty acids.

### Targeted gene expression differences between HFD-fed Cyp2b-null and hCYP2B6-Tg mice as measured by qPCR

**Fig 9:**
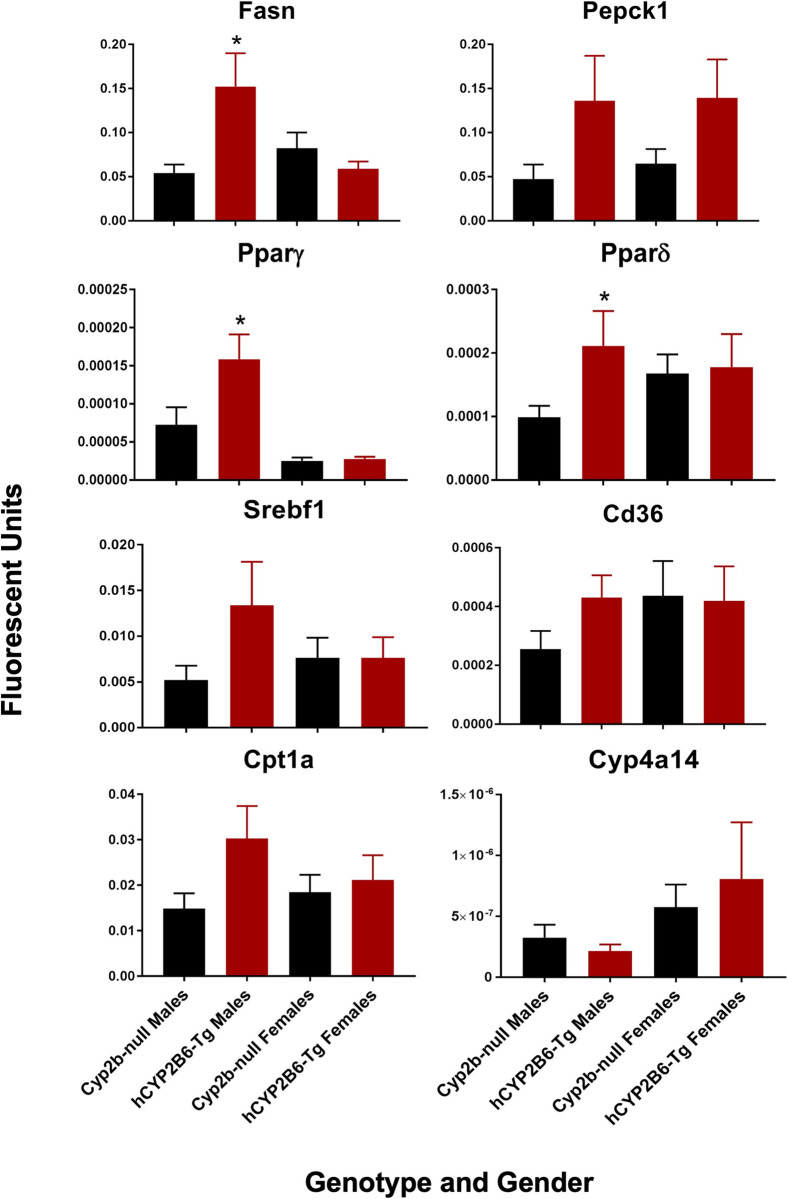
qPCR confirms changes in gene expression of energy metabolism genes regulated by PPARγ in males. Data are presented as mean + SEM. Statistical significance was determinedy by ANOVA followed by Fisher’s LSD as the post-hoc test (n = 5). * indicates a p-value < 0.05.

Changes in the expression of genes involved in energy metabolism (lipid uptake, storage, and metabolism) and primarily regulated by insulin, SREBF1, and PPARs [37, 45] were measured by qPCR. Few of the genes were differentially expressed; however there were some trends in genes primarily regulated by PPARγ including Fasn, Pparγ, Pepck, and Srebf1 [60, 65, 66], of which Fasn and Pparγ were significant (**Fig 9**). Pparδ was also increased as determined by Fisher’s LSD only. Key biomarker genes regulated primarily by PPARα such as Cd36 and Cyp4a14 were not altered [67, 68]. SREBF1 also induces Pparγ and itself similar to PPARγ, but down-regulates Fasn [65, 69]. Therefore, qPCR profiles most closely aligned with activation of PPARγ.

## Discussion

Female hCYP2B6-Tg mice gained less weight than Cyp2b-null counterparts after 16 weeks of HFD treatment. The difference in weight was not as great and the duration of the study was longer than previously conducted when comparing Cyp2b-null and WT mice [10]. This suggests that similar to murine hepatic Cyp2b members, human CYP2B6 is an anti-obesity enzyme but not with the efficacy of the murine Cyp2b enzymes. In addition, the sexual dimorphic effects of human CYP2B6 and murine Cyp2bs were flipped, as Cyp2b-null male mice weighed more than WT mice [10], but it was Cyp2b-null female mice that weighed more than hCYP2B6-Tg mice (**Fig 3**).

In addition to body weight, hCYP2B6-Tg females decreased inguinal WAT mass 27% when compared to Cyp2b-null mice. While 27% is not statistically significant, a 27% drop in WAT mass is biologically impressive. Genes that play a role in adipocyte lipid accumulation, *Angptl8*, [57] and differentiation (*Slc16a1*) [70] were concurrently down-regulated in hCYP2B6-Tg female mice, as well as acyl-coA synthetase short chain family member 2 (*Acss2*), which has been found to promote the systemic storage of fats under HFD conditions in mice [71]. Previous studies have found serum TAG, VLDL and *Angptl8* expression to be positively correlated in humans and mice [72, 73]. These variables were also increased and grouped together by hierarchical cluster analysis in HFD-fed Cyp2b-null female mice. Female RNAseq results also indicate disruption of circadian rhythm genes, which often regulate lipid distribution [74] as well as effects on lipid metabolism/cholesterol/bile acid pathways (*Insig2*) [75] and energy utilization (*Sgk1*) [76]. Several of these genes are consistent with activation of circadian nuclear receptor pathways such as PPARα, PPARγ, and Rev-Erb [61–63, 77, 78], and PPARα and Rev-Erb are directly regulated by BMAL1, a circadian rhythm transcription factor [79].

Concentrations of specific oxylipin metabolites were significantly increased in the serum of hCYP2B6-Tg females, which corresponds well with their signaling role and obesity-associated effects observed in the physiological and transcriptomic results of HFD-fed hCYP2B6-Tg female mice. These altered oxylipin species do not share one specific role, as AA-14,15-EET is anti-inflammatory [80], while LA-9-HODE and 13-HODE are PPARα agonists (**Fig 8**) and as such also potentially anti-inflammatory [81]. Interestingly, 9-HODE and 13-KODE are also agonists of the pain receptor, transient receptor potential vanilloid 1 (TRPV1); 9-HODE, but not 13-HODE, is an agonist for G-protein coupled receptor, G2A/GPR132, and as such would likely be inflammatory [82, 83]. However, TRPV1 is primarily found in the nervous system and skin and unlikely to be involved in actions observed in this study [84].

Hierarchical cluster analysis showed changes in AA-14,15-EET are associated with genes involved in proliferation (*Ccno*, *Frs3*, *Junb*) [85–87]. 9-HODE and 13-KODE (a PPARγ agonist), which are in the same cluster, are associated with genes involved in insulin signaling [88]. Several differentially regulated genes in female hCYP2B6-Tg mice are associated with insulin such as *Irs2*, *Sgk1*, *Inhbb*, *Slc16a1*, *and Angptl8* [89, 90]. This insulin pathway is also known to activate PPARγ [91]. We did not observe activation of PPARγ by 9-HODE or 13-HODE; however, other laboratories have shown evidence that FABP4 is likely induced by a 9-HODE-PPARγ interaction and both 9-HODE and 13-HODE bind the ligand binding pocket of PPARγ [83, 92]. Another study suggests that 13-HODE induces PPARγ activity through repression of PPARδ [93]. We observed activation of PPARγ by 13-KODE (also known as 13-oxo-ODE) at a low concentration of 0.6 μM. Any of these oxylipins may alter insulin signaling or more likely incease PPARγ signaling. Ultimately, these changes in hepatic gene expression and serum oxylipin concentrations between HFD-fed hCYP2B6-Tg and Cyp2b-null female mice suggest CYP2B6 could affect lipid distribution.

Although AA and DHA had the lowest IC50 values compared to the other PUFAs measured by concentration-dependent response curves, ALA was the predominant substrate metabolized by CYP2B6 *in vitro* followed by LA and AA when adequate substrate was provided. CYP2B6 oxylipin metabolites were produced in the 9- and 13- positions at high concentrations, especially 9-HOTrE followed by 13-HOTrE. The oxylipin 13-HOTrE is known for its role in suppression of inflammation [94, 95], and although the biological effect of the previously determined lipoxygenase product 9-HOTrE [74] is not established, it is predicted to share a similar anti-inflammatory role [96].

Conversely, important or significantly altered PUFA metabolites from liver and serum samples of HFD-fed hCYP2B6-Tg mice suggests CYP2B6 primarily metabolizes the n-6 PUFAs, LA and AA *in vivo*, in the 9- and 13- positions. This change in substrate preference compared to the *in vitro* data is most likely attributed to the available concentrations of the different PUFAs and their affinity for CYP2B6 (**Fig 1**). The HFD provided to the mice has nearly 15X more n-6 than n-3 PUFAs and the main source of PUFAs in the HFD treatment was soybean oil, which is approximately 55% LA and only 7% ALA [97]. In addition, ALA had a slightly lower affinity to CYP2B6 compared to AA according to concentration-dependent response curve results, indicating more ALA would need to be present for metabolism to occur. Overall, several oxylipins are produced from CYP2B6 and preferentially present in hCYP2B6-Tg mice, indicating that CYP2B6, at least under high-fat conditions, metabolizes PUFAs to several oxylipins with preference in the 9- and 13-positions.

In contrast to females, there was no change in weight between genotypes in HFD-fed male mice, although male hCYP2B6-Tg mice exhibited increased glucose sensitivity and higher liver triglyceride levels compared to Cyp2b-null males. High fat diets often cause a shift from normal triglyceride synthesis to bioactive lipid intermediates that can induce endoplasmic reticulum (ER) stress and cause lipotoxicity [98]; however, lipotoxic ER stress was not observed in male hCYP2B6-Tg mice. We suspect that the increase in inert hepatic triglycerides provided lipotoxic protection from other fatty acid-derived species [99]. Additionally, the downregulation of *Egfr* in male hCYP2B6-Tg provides a mechanism for the up-regulation of Cyp2b10 through CAR activation [58] and potentially explains the physiological and lipid metabolism differences between male and female hCYP2B6-Tg mice, as *Egfr* was up-regulated in females.

Differences are also potentially due to the differing roles of human versus murine CYP2B, as CAR activation in murine models inhibits gluconeogenesis, lipogenesis and fatty acid synthesis, but in human hepatocytes CAR was only found to inhibit gluconeogenesis [100], and RNAseq suggests the potential for CAR activation based on decreased *Egfr* and *Cyp7a1*, and increased CYP2B6/*Cyp2b10* [58], as well as *gstm3*, *sgpl1*, *por*, *sdf2l1*, *insig2*, *hspb1*, *and sgk1* [51]. Interestingly, decreased *Egfr* and *Cyp7a1* were associated with decreased serum cholesterol and HDL in hCYP2B6-Tg males. Furthermore, the most important oxylipin in the livers of HFD-fed hCYP2B6-Tg mice as determined by random forest, AA-14,15-EET, and other CYP-derived EETs contribute positively to insulin sensitivity [101]. Taken together, there appears to be several pieces of evidence that suggest a positive role for CYP2B6 in glucose tolerance.

Observed differences between male hCYP2B6-Tg and male Cyp2b-null mice were predominantly liver-based effects such as increased liver triglycerides and a change in glucose tolerance. HFD-fed hCYP2B6-Tg males also presented with increased total average concentrations of hepatic oxylipin species compared to their Cyp2b-null counterparts with PPARα and PPARγ activators, 9-HODE and 13-KODE considered key increased oxylipins as determined by Random Forest (**Fig 5**). Increased levels of oxylipin metabolites of ALA and LA have been previously associated with soybean oil-induced fatty liver and obesity in mice [102]. Similar to females, hCYP2B6-Tg male mice also experienced perturbations in circadian rhythm-associated genes that are important mediators of hepatic lipid homeostasis [56].

In humans, excess intrahepatic fat and visceral adipose tissue (VAT) have been associated with perturbed glucose and lipid metabolism. VAT is highly lipolytic and increases levels of free fatty acids in the liver, causing enhanced gluconeogenesis and hepatic insulin resistance [103]. It has been shown that adults and children with NAFLD have impaired glucose tolerance in equal proportions to the degree of steatosis [104]. However, in this study HFD-fed hCYP2B6-Tg male mice simultaneously increased hepatic triglycerides and glucose tolerance compared to Cyp2b-null mice, which is unusual but not unprecedented [105] as other studies have shown that acute NAFLD can be protective [106].

PPARγ activation is one of the few mechanisms that can explain an association between increased liver steatosis and improved glucose tolerance. PPARγ activation in diabetic patients improves whole-body insulin sensitivity [107] at the same time increasing steatosis [108]. Therefore, targeting PPAR for improving diabetes comes with conern for potential side effects allowing for greater NAFLD [108]. In this study, we observed increased liver triglycerides along with improved glucose tolerance in the hCYP2B6-Tg mice in association with increased oxylipins that activate PPARs and changes in gene expression associated with PPAR activation. qPCR confirmation of key biomarker genes showed increased Pparγ and Fasn expression with no change in Cyp4a14 and Cd36 expression, indicating PPARγ activation; and not likely PPARα activation. Overall, our data is consistent with CYP2B6 expression leading to increased oxylipins that are associated with increased PPARγ activity and improved glucose sensitivity at the cost of increased steatosis.

Differences are also potentially due to the differing roles of human versus murine CYP2B, as CAR activation in murine models inhibits gluconeogenesis, lipogenesis and fatty acid synthesis, but in human hepatocytes CAR was only found to inhibit gluconeogenesis [100], and RNAseq suggests the potential for CAR activation based on decreased *Egfr* and *Cyp7a1*, and increased CYP2B6/*Cyp2b10* [58], as well as *gstm3*, *sgpl1*, *por*, *sdf2l1*, *insig2*, *hspb1*, *and sgk1* [51]. Interestingly, decreased *Egfr* and *Cyp7a1* were associated with decreased serum cholesterol and HDL in hCYP2B6-Tg males. Furthermore, the most important oxylipin in the livers of HFD-fed hCYP2B6-Tg mice as determined by random forest, AA-14,15-EET, and other CYP-derived EETs contribute positively to insulin sensitivity [101]. Taken together, there appears to be several pieces of evidence that suggest a positive role for CYP2B6 in glucose tolerance.

In conclusion, the data presented indicates CYP2B6 is an anti-obesity enzyme in humanized mice, which verifies epidemiological data [3]. CYP2B6 metabolizes PUFAs *in vitro* and *in vivo* preferentially in the 9- and 13- positions on LA and ALA, with more limited metabolism of AA and DHA. Previous research indicates that several of these oxylipins are anti-inflammatory mediators of metabolic disease, however some are inflammatory oxylipins. In addition, several of the CYP2B6-produced 9- and 13-position oxylipins are PPARα and PPARγ activators, providing a putative mechanism for CYP2B6 as an anti-obesity enzyme. HFD-fed hCYP2B6-Tg male and female mice were less susceptible to the development of metabolic disease compared to Cyp2b-null mice through different mechanisms as female mice showed reduced body weight and males increased glucose sensitivity consistent with PPARγ activity. Overall, this study provides a putative mechanism by which CYP2B6 acts as an anti-obesity/anti-metabolic disease enzyme under HFD conditions and suggests how chemical inhibition or polymorphic loss of CYP2B6 activity could increase diet-induced obesity and metabolic disease through reduced production of important oxylipins or changes in circadian-mediated regulation of lipid metabolism and distribution.

## Supporting information

S1 File Feed consumption of Cyp2b-null and hCYP2B6-Tg mice during 16-weeks of high-fat diet treatment. Female and male feed consumption was measured by weighing the food every alternate day. Data are presented as mean calories ± SEM. Statistical significance was determined by unpaired Student’s t-tests (n = 8). * indicates a p-value < 0.05.
(PDF)

S2 File Tabular comparison of tissue weights between Cyp2b-null and hCYP2B6-Tg female (A) and male (B) mice fed a HFD for 16 weeks. (PDF)

S3 File Tabular comparison of serum biomarkers between Cyp2b-null and hCYP2B6-Tg female (A) and male (B) mice fed a HFD for 16 weeks. (PDF)

S4 File Table of total average of oxylipin metabolites measured in liver and serum of HFD-fed Cyp2b-null and hCYP2B6-Tg mice. (PDF)

S5 File Table containing measured serum and liver lipid metabolite concentrations in Cyp2b-null and hCYP2B6-Tg female (A) and male (B) mice fed a HFD for 16 weeks. (PDF)

S6 File Graphical representation of increased oxylipins in serum of female HFD-fed hCYP2b6-Tg mice. Serum oxylipins in female and male serum. Data are presented as mean ± SEM. Statistical significance was determined by unpaired Student’s t-tests (n = 4–5). * indicates a p-value < 0.05 and ** indicates a p-value < 0.01.
(PDF)

S7 File Differentially expressed gene lists of female and male HFD-fed hCYP2B6-Tg mice compared to HFD-fed Cyp2b-null mice, GO term enrichment, Enrichr data, organ weights, serum lipids, Cyp2b inhibition, and oxylipin production. (XLSX)

S8 File CYP2B6 alignment to Cyp2b10 on mouse reference genome. Alignment of male hCYP2B6-Tg bam files to mouse reference genome using IGV viewer. The high number single nucleotide polymorphisms (SNPs) within the exon regions of Cyp2b10 is provides evidence of the human CYP2B6 misalignment to mouse Cyp2b10.
(PDF)
