# Supplementary material for: Human CYP2B6 produces oxylipins from polyunsaturated fatty acids and reduces diet-induced obesity
Source: PLoS One. 2022 Dec 15;17(12):e0277053. doi: 10.1371/journal.pone.0277053 (PMC9754190; doi:10.1371/journal.pone.0277053)
Supplement: S1 File — Female and male feed consumption was measured by weighing the food every alternate day. Data are presented as mean calories ± SEM. Statistical significance was determined by unpaired Student’s t-tests (n = 8). * indicates a p-value < 0.05. (PDF) [file pone.0277053.s001.pdf]

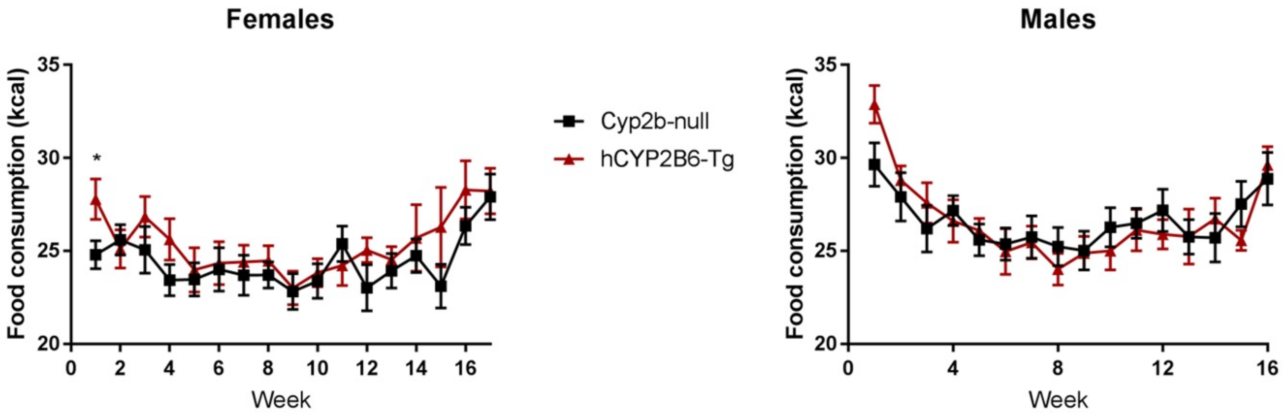

Supplementary File 1. Feed consumption of Cyp2b-null and hCYP2B6-Tg mice during 16-weeks of high-fat diet treatment. Female and male feed consumption was measured by weighing the food every alternate day. Data are presented as mean calories ± SEM. Statistical significance was determined by unpaired Student's t-tests (n=8). * indicates a p-value < 0.05.
