# Supplementary material for: Human CYP2B6 produces oxylipins from polyunsaturated fatty acids and reduces diet-induced obesity
Source: PLoS One. 2022 Dec 15;17(12):e0277053. doi: 10.1371/journal.pone.0277053 (PMC9754190; doi:10.1371/journal.pone.0277053)
Supplement: S2 File — (PDF) [file pone.0277053.s002.pdf]

Suppl File 2. Comparison of tissue weights between Cyp2b-null and hCYP2B6-Tg female (A) and male (B) mice fed a HFD for 16 weeks.

A.

**Tissue Weights**	**Cyp2b-null F**	**hCYP2B6-Tg F**
**Total Body**	29.63 ± 1.40	27.05 ± 1.21
**Liver**	1.02 ± 0.03	0.99 ± 0.04
**Kidney**	0.33 ± 0.01	0.29 ± 0.01*
**WAT**	2.25 ± 0.33	1.65 ± 0.31
**BAT**	0.13 ± 0.02	0.12 ± 0.01

B.

**Tissue Weights**	**Cyp2b-null M**	**hCYP2B6-Tg M**
**Total Body**	38.81 ± 1.99	38.93 ± 1.66
**Liver**	1.40 ± 0.13	1.51 ± 0.07
**Kidney**	0.48 ± 0.04	0.52 ± 0.02
**WAT**	3.41 ± 0.07	3.52 ± 0.33
**BAT**	0.31 ± 0.03	0.29 ± 0.07
**Testes**	0.33 ± 0.03	0.38 ± 0.03

Data are presented as mean (g) ± SEM. Statistical significance was determined by unpaired Student's t-tests (n=7-8). * indicates a p-value < 0.05.
