# Supplementary material for: Human CYP2B6 produces oxylipins from polyunsaturated fatty acids and reduces diet-induced obesity
Source: PLoS One. 2022 Dec 15;17(12):e0277053. doi: 10.1371/journal.pone.0277053 (PMC9754190; doi:10.1371/journal.pone.0277053)
Supplement: S3 File — (PDF) [file pone.0277053.s003.pdf]

Suppl File 3. Comparison of serum biomarkers between Cyp2b-null and hCYP2B6-Tg female (A) and male (B) mice fed a HFD for 16 weeks.

A.

Serum Biomarkers	Cyp2b-null F	hCYP2B6-Tg F
**ALT**	21.76 ± 2.02	18.81 ± 1.14
**Triglycerides**	87.53 ± 15.68	71.33 ± 12.36
**HDL**	74.37 ± 2.88	75.26 ± 1.78
**LDL**	6.75 ± 0.46	7.59 ± 0.29
**VLDL**	17.51 ± 3.14	14.27 ± 2.47
**Cholesterol**	133.24 ± 7.44	127.82 ± 4.05

B.

Serum Biomarkers	Cyp2b-null M	hCYP2B6-Tg M
**ALT**	40.02 ± 15.89	30.65 ± 7.51
**Triglycerides**	88.06 ± 9.61	79.25 ± 7.31
**HDL**	100.76 ± 3.03	98.70 ± 1.76
**LDL**	10.90 ± 0.76	10.97 ± 0.76
**VLDL**	17.61 ± 1.92	15.85 ± 1.46
**Cholesterol**	213.71 ± 6.18	200.52 ± 6.56

Data are presented as mean (g) ± SEM. Statistical significance was determined by unpaired Student’s t-tests (n=5).
