# Supplementary material for: Human CYP2B6 produces oxylipins from polyunsaturated fatty acids and reduces diet-induced obesity
Source: PLoS One. 2022 Dec 15;17(12):e0277053. doi: 10.1371/journal.pone.0277053 (PMC9754190; doi:10.1371/journal.pone.0277053)
Supplement: S4 File — (PDF) [file pone.0277053.s004.pdf]

Suppl File 4. Total average of oxylipin metabolites measured in liver and serum of HFD-fed Cyp2b-null and hCYP2B6-Tg mice.

	**Serum**		**Liver**	
	**Cyp2b-null**	**hCYP2B6-Tg**	**Cyp2b-null**	**hCYP2B6-Tg**
**Females**	0.002521 ± 0.0005892	0.003214 ± 0.0007361	0.007582 ± 0.001603	0.004072 ± 0.000926
**Males**	0.002238 ± 0.0005311	0.005842 ± 0.002113	0.002008 ± 0.0004811	0.004106 ± 0.0008813*

Data are presented as mean (ng/µL) ± SEM. Statistical significance was determined by unpaired Student's t-tests (n=5). * indicates a p-value < 0.05.
