# Supplementary material for: Human CYP2B6 produces oxylipins from polyunsaturated fatty acids and reduces diet-induced obesity
Source: PLoS One. 2022 Dec 15;17(12):e0277053. doi: 10.1371/journal.pone.0277053 (PMC9754190; doi:10.1371/journal.pone.0277053)
Supplement: S5 File — (PDF) [file pone.0277053.s005.pdf]

Suppl File 5 Measured serum and liver lipid metabolite concentrations in Cyp2b-null and hCYP2B6-Tg female (A) and male (B) mice fed a HFD for 16 weeks.
A.

	Serum		Liver	
**Oxylipin Species**	**Cyp2b-null**	**hCYP2B6-Tg**	**Cyp2b-null**	**hCYP2B6-Tg**
**AA 14,15-EET**	3.444E-06 ± 2.11E-06	2.909E-05 ± 2.946E-06**	0.0008699 ± 0.0004062	0.0003727 ± 0.000219
**AA 11,12-EET**	0.001911 ± 0.0003876	0.002914 ± 0.001033	0.007769 ± 0.002561	0.003422 ± 0.001977
**AA 9-HETE**	0.008382 ± 0.005741	0.007522 ± 0.003799	0.01077 ± 0.005417	0.006018 ± 0.002752
**AA 12-HETE**	0.0004833 ± 9.685E-05	0.001130 ± 0.0003599	0.002732 ± 0.0008528	0.001756 ± 0.001041
**AA 11,12-DHET**	0.01543 ± 0.003179	0.02971 ± 0.006977	0.02317 ± 0.009411	0.01133 ± 0.00699
**AA PGE2**	0.0047044 ± 0.001773	0 ± 0*	0.0002838 ± 0.0002838	0 ± 0
**AA PGF2a**	0.003416 ± 0.0008837	0.005472 ± 0.001738	0.04234 ± 0.02609	0.02244 ± 0.01324
**AA TXB2**	6.141E-05 ± 2.902E-05	0.0001025 ± 4.572E-05	0.001356 ± 0.000447	0.0006450 ± 0.0004188
**LA 9-HODE**	0.001124 ± 0.0001462	0.004251 ± 0.0009221*	0.03462 ± 0.0141	0.01603 ± 0.009448
**LA 9-HpODE**	0.02914 ± 0.002886	0.02957 ± 0.004296	0.03637 ± 0.01197	0.03278 ± 0.004932
**LA 13-KODE**	0.0003835 ± 6.357E-05	0.001051 ± 0.0001858**	0.007301 ± 0.00389	0.002536 ± 0.001444
**LA 9,10-DiHOME**	9.894E-05 ± 4.307E-05	0.000345 ± 8.309E-05*	0.002194 ± 0.0008728	0.0008417 ± 0.0005167
**EPA Resolvin**	0.002928 ± 0.0001994	0.004320 ± 0.0006148	0.0007463 ± 0.0004635	0.0003922 ± 0.0002707
**ALA 9-HOTrE**	0 ± 0	0.0001253 ± 0.0001253	0.0006108 ± 0.0002531	0.0002325 ± 0.0002325
**ALA Isoprostane**	0 ± 0	0.0002449 ± 6.423E-05**	0.008005 ± 0.003746	0.003327 ± 0.001886

B.

	Serum		Liver	
**Oxylipin Species**	**Cyp2b-null**	**hCYP2B6-Tg**	**Cyp2b-null**	**hCYP2B6-Tg**
**AA 14,15-EET**	1.281E-05 ± 4.445E-06	3.707E-05 ± 2.216E-05	8.732E-05 ± 2.825E-05	0.0003897 ± 0.0002161
**AA 11,12-EET**	0.002183 ± 0.0006091	0.006343 ± 0.003742	0.0009780 ± 0.0003435	0.004964 ± 0.002834
**AA 9-HETE**	0.003868 ± 0.001342	0.01030 ± 0.003904	0.006016 ± 0.001504	0.01118 ± 0.003558
**AA 12-HETE**	0.0004491 ± 0.0001189	0.002130 ± 0.001214	0.0006437 ± 0.000258	0.002592 ± 0.001547
**AA 11,12-DHET**	0.01763 ± 0.00509	0.04130 ± 0.02508	0.002351 ± 0.001458	0.01177 ± 0.006342
**AA PGE2**	0.002081 ± 0.0008876	0.002618 ± 0.001158	0.0004154 ± 0.0004154	0 ± 0
**AA PGF2a**	0.001286 ± 0.0008909	0.007236 ± 0.003804	0.005866 ± 0.002478	0.02343 ± 0.01314
**AA TXB2**	0 ± 0	0.0001574 ± 5.767E-05*	0.0002758 ± 0.0001044	0.00140 ± 0.0008361
**LA 9-HODE**	0.001534 ± 0.0005044	0.004277 ± 0.00226	0.004474 ± 0.001505	0.01805 ± 0.01013
**LA 9-HpODE**	0.02545 ± 0.002158	0.02385 ± 0.007709	0.02616 ± 0.00340	0.02363 ± 0.004619
**LA 13-KODE**	0.0005594 ± 0.0001023	0.001398 ± 0.0008135	0.0006725 ± 0.0001771	0.002463 ± 0.001289
**LA 9,10-DiHOME**	9.070E-05 ± 4.131E-05	0.0002486 ± 0.0001336	0.0003869 ± 0.000138	0.001156 ± 0.0006744
**EPA Resolvin**	0.003274 ± 0.000697	0.003091 ± 0.0008124	0 ± 0	0 ± 0
**ALA 9-HOTrE**	0 ± 0	0 ± 0	0 ± 0	0.0004337 ± 0.000286
**ALA Isoprostane**	0.0001335 ± 5.946E-05	0.0003280 ± 0.000126	0.0008786 ± 0.000269	0.003881 ± 0.002122

Data are presented as mean (ng/µL) ± SEM. Statistical significance was determined by unpaired Student's t-tests (n=5). * indicates a p-value < 0.05 and ** indicates a p-value < 0.01.
