# Supplementary material for: Human CYP2B6 produces oxylipins from polyunsaturated fatty acids and reduces diet-induced obesity
Source: PLoS One. 2022 Dec 15;17(12):e0277053. doi: 10.1371/journal.pone.0277053 (PMC9754190; doi:10.1371/journal.pone.0277053)

Suppl File 6: Graphical representation of increased oxylipins in serum of female HFD-fed hCYP2b6-Tg mice

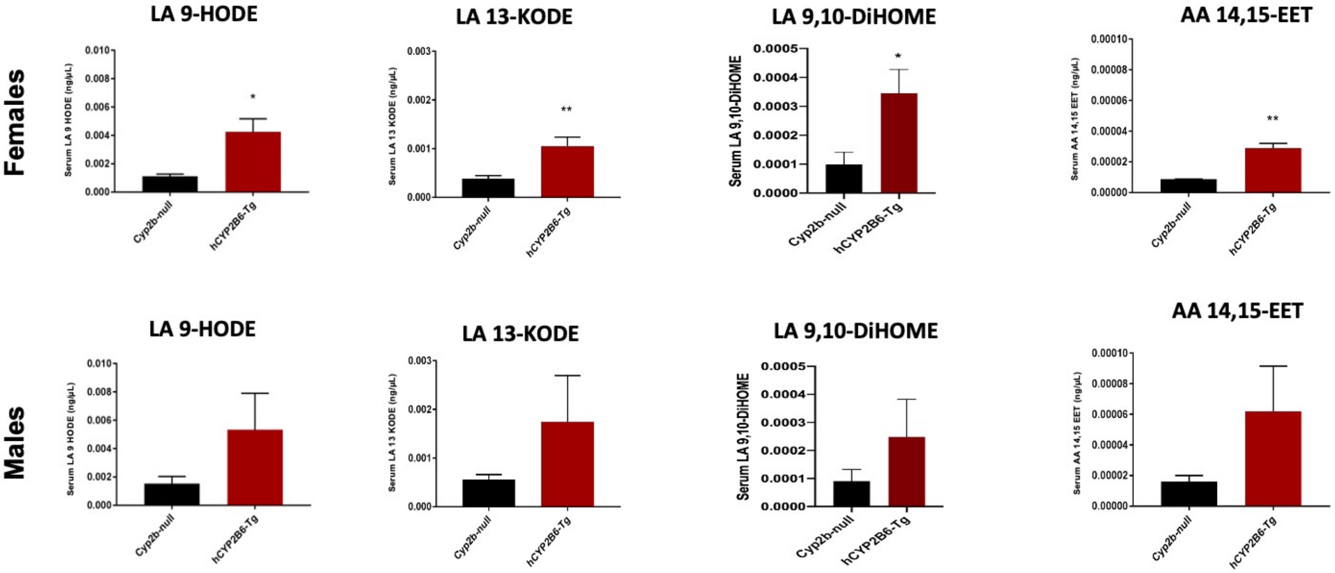

Supplement: S6 File — Serum oxylipins in female and male serum. Data are presented as mean ± SEM. Statistical significance was determined by unpaired Student’s t-tests (n = 4–5). * indicates a p-value < 0.05 and ** indicates a p-value < 0.01. (PDF) [file pone.0277053.s006.pdf]
