# Supplementary material for: Human CYP2B6 produces oxylipins from polyunsaturated fatty acids and reduces diet-induced obesity
Source: PLoS One. 2022 Dec 15;17(12):e0277053. doi: 10.1371/journal.pone.0277053 (PMC9754190; doi:10.1371/journal.pone.0277053)
Supplement: S8 File — Alignment of male hCYP2B6-Tg bam files to mouse reference genome using IGV viewer. The high number single nucleotide polymorphisms (SNPs) within the exon regions of Cyp2b10 is provides evidence of the human CYP2B6 misalignment to mouse Cyp2b10. (PDF) [file pone.0277053.s008.pdf]

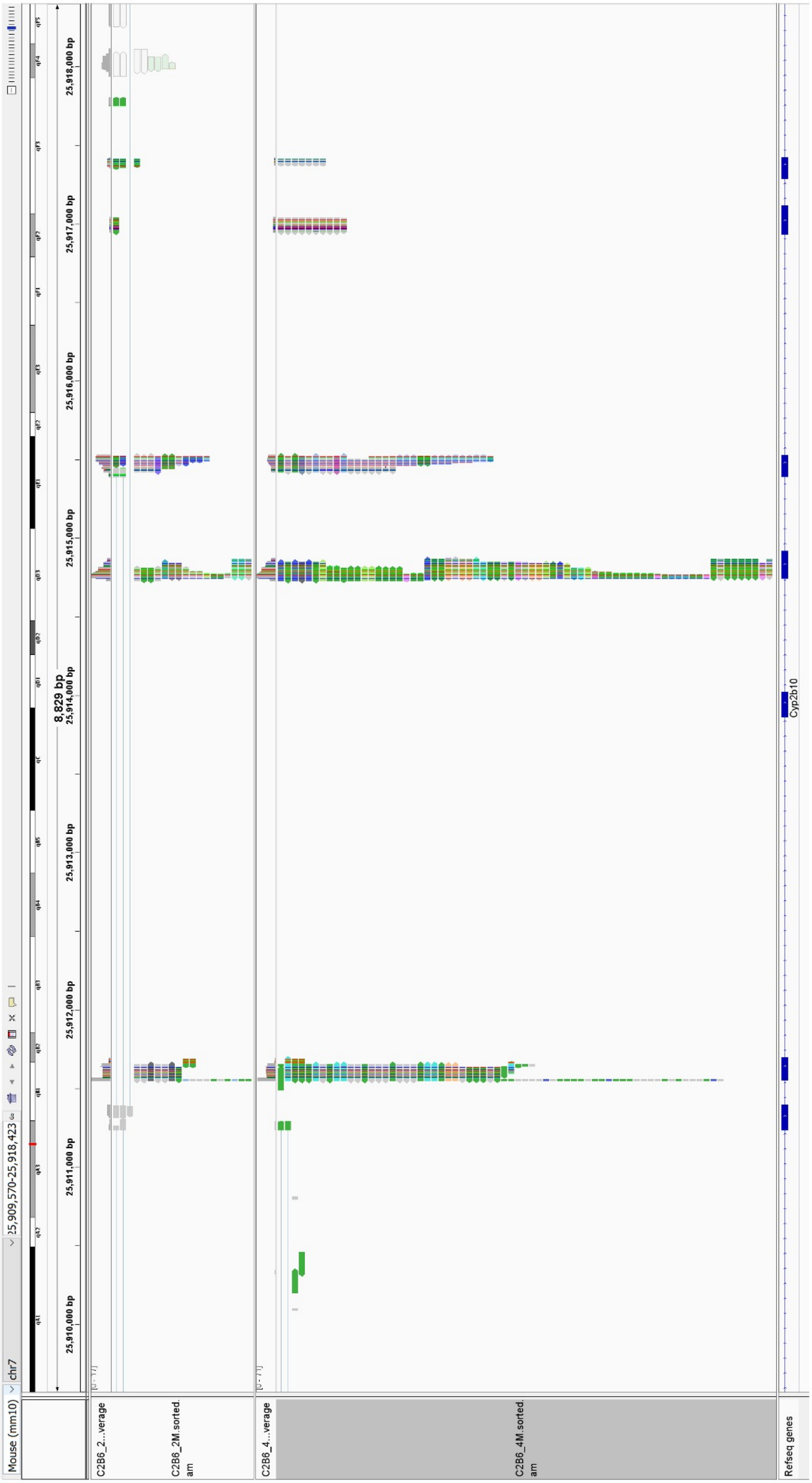

Supplementary File 8. CYP2B6 alignment to Cyp2b10 on mouse reference genome. Alignment of male hCYP2B6-Tg bam files to mouse reference genome using IGV viewer (72). The high number single nucleotide polymorphisms (SNPs) within the exon regions of Cyp2b10 is provides evidence of the human CYP2B6 misalignment to mouse Cyp2b10.
